# Genome sequencing of the high oil crop sesame provides insight into oil biosynthesis

**DOI:** 10.1186/gb-2014-15-2-r39

**Published:** 2014-02-27

**Authors:** Linhai Wang, Sheng Yu, Chaobo Tong, Yingzhong Zhao, Yan Liu, Chi Song, Yanxin Zhang, Xudong Zhang, Ying Wang, Wei Hua, Donghua Li, Dan Li, Fang Li, Jingyin Yu, Chunyan Xu, Xuelian Han, Shunmou Huang, Shuaishuai Tai, Junyi Wang, Xun Xu, Yingrui Li, Shengyi Liu, Rajeev K Varshney, Jun Wang, Xiurong Zhang

**Affiliations:** 1 Oil Crops Research Institute of the Chinese Academy of Agricultural Sciences, Key Laboratory of Biology and Genetic Improvement of Oil Crops of the Ministry of Agriculture, Wuhan 430062, China; 2 Beijing Genomics Institute (BGI) - Shenzhen, Shenzhen 518083, China; 3 Department of Biology, University of Copenhagen, Copenhagen DK-2200, Denmark; 4 Yanzhuang Oil Co., Ltd, Hefei 231283, China; 5 International Crops Research Institute for the Semi-Arid Tropics (ICRISAT), Patancheru 502324, India; 6 CGIAR Generation Challenge Programme (GCP), c/o CIMMYT, Mexico DF 6-641 06600, Mexico

## Abstract

**Background:**

Sesame, *Sesamum indicum* L., is considered the queen of oilseeds for its high oil content and quality, and is grown widely in tropical and subtropical areas as an important source of oil and protein. However, the molecular biology of sesame is largely unexplored.

**Results:**

Here, we report a high-quality genome sequence of sesame assembled *de novo* with a contig N50 of 52.2 kb and a scaffold N50 of 2.1 Mb, containing an estimated 27,148 genes. The results reveal novel, independent whole genome duplication and the absence of the Toll/interleukin-1 receptor domain in resistance genes. Candidate genes and oil biosynthetic pathways contributing to high oil content were discovered by comparative genomic and transcriptomic analyses. These revealed the expansion of type 1 lipid transfer genes by tandem duplication, the contraction of lipid degradation genes, and the differential expression of essential genes in the triacylglycerol biosynthesis pathway, particularly in the early stage of seed development. Resequencing data in 29 sesame accessions from 12 countries suggested that the high genetic diversity of lipid-related genes might be associated with the wide variation in oil content. Additionally, the results shed light on the pivotal stage of seed development, oil accumulation and potential key genes for sesamin production, an important pharmacological constituent of sesame.

**Conclusions:**

As an important species from the order Lamiales and a high oil crop, the sesame genome will facilitate future research on the evolution of eudicots, as well as the study of lipid biosynthesis and potential genetic improvement of sesame.

## Background

Sesame (*Sesamum indicum*), a widely grown crop in tropical and subtropical areas, is documented as the most ancient oil crop providing humans with essential daily energy. Vegetable oil consumption is expected to reach almost 200 billion kilograms by 2030 [1], which will increase the demand for oil-rich crops; genetic studies to improve oil content in vegetables will help address this demand. Compared to other edible oil crops such as soybean (*Glycine max*), rapeseed (*Brassica napus*), peanut (*Arachis hypogaea*) and olive (*Olea europaea*), sesame has innately higher oil content (approximately 55% of dry seed) [2], and is thus an attractive potential model for studying lipid biosynthesis [3].

The sesame seed has been considered the ‘queen of oilseeds’ for its high oil content and quality [4], and has been traditionally categorized as a health food in China, Japan and other East Asian countries [5]. The antioxidative furofuran lignans in sesame have been analyzed by pharmacologists for their potent pharmacological properties in decreasing blood lipids [6] and lowering cholesterol levels [7]. Recently, the gene encoding sesamin synthase was identified [8].

Taxonomically, sesame belongs to Lamiales, an order comprising of 23,810 flowering plants in the clade asterids [9]. Lamiales includes many other well-known or economically important species, such as olive (*O. europaea*), leonurus (*Leonurus japonicas*), lavender (*Lavandula spica*) and basil (*Ocimum basilicum*). However, few Lamiales species have been the subject of intensive genetic or genomics studies. As high-throughput sequencing has become routine, several studies in sesame have been performed. A number of simple sequence repeat (SSR) markers have been developed [10-12]. The loci associated with indehiscent capsule trait [13], determinate growth habit [14] and seed coat color [15] have been detected, and the expression levels of sesame genes have been explored using Sanger and high-throughput DNA sequencing technologies [16-18]. The phylogenetic position of sesame has been determined using chloroplast genomic data, which indicated the core lineage of *Sesamum* in Lamiales [19]. These studies together with the recently published minute genome of *Utricularia gibba*[20] and a genome survey of sesame [21] have contributed new insight into the Lamiales.

Here, we report a high-quality draft genome of the sesame genotype ‘Zhongzhi No. 13’ , an elite cultivar with high oil content (59%), which has been introduced to most major sesame planting areas in China over the past 10 years. In addition to the well-assembled genome sequences, a new high-density genetic map, 12 in-depth RNA-Seq data sets and resequencing data for 29 sesame accessions were generated to help understand and analyze genome structure, evolution and important nutritional characters, including lipid and sesamin synthesis, in the most comprehensive way. Together, these results will open a door for genetic studies for a variety of purposes in, but not restricted to, sesame.

## Results and discussion

### *De novo* genome sequencing

#### Assembly and assessment

**Figure 1:**
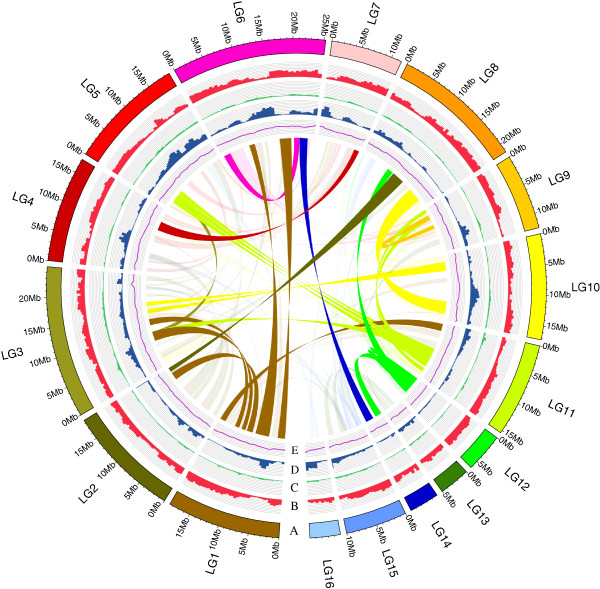
**Distribution of the basic genomic elements of sesame. (A)** Pseudomolecules. **(B)** Gene density (mRNA); the frequency of sites within gene regions per 500 kb ranged from 0.04 to 0.61. **(C)** DNA transposon element (TE) density; the frequency of sites within DNA TE regions per 500 kb ranged from 0 to 0.22. **(D)** Retrotransposon element density; the frequency of sites per 500 kb within retrotransposon element regions ranged from 0 to 0.71. **(E)** GC content; the ratio of GC sites per 100 kb ranged from 0.32 to 0.40. Inner ribbons indicate self-collinearity of sesame, and the homologous regions of more than 1 Mb are highlighted. Circos [24] was used to construct the diagram.

After reads filtering, 54.5 Gb of high-quality data from sesame cultivar ‘Zhongzhi No. 13’ were obtained using the Illumina Hiseq2000 platform (Figure S1 and Tables S1 and S2 in Additional file 1; Data S1 in Additional file 2), representing approximately 152.7-fold coverage of the predicted sesame genome. SOAPdenovo [22] was used to assemble the genome, which resulted in a draft genome of 274 Mb with contig N50 of 52.2 kb and scaffold N50 of 2.1 Mb, which are approximately 2.7- and 92.9-fold longer, respectively, than a previous survey of the sesame genome [21] (Table 1; Tables S3 and S4 in Additional file 1). Using a newly constructed genetic map consisting of 406 markers (Data S2 in Additional file 2), we anchored 150 large scaffolds (117 oriented) into 16 pseudomolecules, which harbored 85.3% of the genome assembly and 91.7% of the predicted genes (numbered LG1 to LG16; Table 1; Figure 1; Table S5 and Figures S3 to S5 in Additional file 1). The estimated heterozygosity of the assembled sequenced line was 1.08 × 10^−4^. This low heterozygosity was not unexpected because sesame is a self-pollinated plant [23], and we had performed successive selfing for five generations on the sample before sequencing to guarantee its homozygosity.

**Table 1:** Summary of sesame genome assembly and annotation

**Assembly**		**Number**	**N50 (size/number)**	**N90 (size/number)**	**Total length**
Contigs	All	26,239	52.17 kb/1,545	11.40 kb/5,534	270 Mb
Scaffolds	All	16,444	2.10 Mb/42	268.23 kb/169	274 Mb
	Anchored on chromosomes	150	-	-	234 Mb
	Anchored on chromosomes and oriented	117	-	-	207 Mb
Annotation		Number	Total length	Percentage of the assembly	
Protein coding genes	All	27,148	86.08 Mb	31.46	
Transposable elements	All	-	78.86 Mb	28.46	
	LTR retroelements	-	48.03 Mb	17.56	
	Non-LTR retrotransposons^a^	-	11.70 Mb	4.28	
	DNA transposons	-	10.88 Mb	3.98	
	Unknown	-	14.64 Mb	5.35	
Non-coding RNAs	rRNA fragments	386	89.66 kb	<0.04	
	tRNAs	870	65.31 kb	<0.03	
	miRNAs	207	25.41 kb	<0.01	
	snRNAs	268	33.93 kb	<0.02	

^a^Long interspersed nuclear elements (LINEs) and short interspersed nuclear elements (SINEs). LTR, long terminal repeats; miRNA, microRNA; snRNA, small nuclear RNA.

The assembly covered 77.4 to 81.3% of the genome size according to the estimations derived from 17-mer depth distribution (357 Mb) (Figure S2 in Additional file 1) and flow cytometry (337 Mb) (Figure S3 in Additional file 1). The integrity of gene space in the genome assembly was demonstrated by the successful mapping of 99.3% of 3,328 expressed sequence tags (ESTs) [16] retrieved from GenBank, and 98.5% of 86,222 unigenes that were assembled *de novo* from previously reported RNA-Seq data [17] (Table S6 in Additional file 1). In addition, the large-scale assembly accuracy was assessed using five fosmid clones (33.5 to 38.6 kb) that were sequenced thoroughly using the Sanger sequencing technology (see Supplementary Note in Additional file 1), whereby 99.6% of the clone sequences, on average, were identical to the assembly (Table S7 and Figure S7 in Additional file 1). Hence, a high-quality assembly of sesame is provided here, rendering it a valuable source for studying genome structure and evolution.

#### Genome annotation

We predicted 27,148 protein-coding genes with an average transcript size of 3,171 bp by *ab initio* and homology-based analyses (Tables S8 to S10 in Additional file 1), together with RNA-Seq reads-assisted annotation. Of those, 23,635 (87.1%) were supported by unigenes or protein similarity, with only 12.9% derived solely from *ab initio* gene predictions (Table S9 in Additional file 1). With regard to non-coding genes, we identified 207 microRNAs, 870 tRNAs, 268 small nuclear RNAs, and 386 rRNA fragments from the assembly (Table S11 in Additional file 1).

Transposable elements play an important role in and are the major components of plants. A comprehensive annotation revealed that the repeat elements in sesame are lower (28.5% of assembly) than in grapevine (52.2%) [25], tomato (63.2%) [26] and potato (54.5%) [27] (Table 1; Tables S12 and S13 in Additional file 1). As observed in other sequenced genomes, long terminal repeats (LTRs) in sesame occupy the majority (51.1%) of repeat sequences. When the number of full-length LTRs (*Copia* and *Gypsy*) was plotted against their insertion time inferred from the intra-sequence divergence of LTR regions (Figure S8 in Additional file 1), the resulting age distributions exhibited typical ‘L’ shapes with the accumulation of many recent LTRs and much fewer old LTRs. These age distributions reflected a steady-state stochastic birth/death model for the dynamics of LTR accumulation and activity [28].

For the two major members of LTRs, the proportion of *Copia* (7.3% of the genome) in sesame is comparable to that in grapevine, tomato and potato, but the percentage of *Gypsy* (6.6%) is extremely underrepresented (Table S13 in Additional file 1). Unlike that observed in tomato and potato, the distribution of the divergence rate of *Gypsy* is very smooth and low in sesame, suggesting that it had not experienced explosive accumulation or activity (Figure S9 in Additional file 1), and may be associated with the low proportion of *Gypsy*, which in turn relates to the lower repeat element ratio and smaller genome size of sesame.

### Evolution in the sesame genome

#### Gamma and a recent whole genome duplication event in sesame

Sesame belongs to the asterids clade of eudicots [29]. Taxonomically, it is mostly related to *Utricularia gibba*[20], tomato [26] and potato [27], the other whole genome sequences available and published thus far in this clade. Based on the shared single-copy genes from 11 sequenced species (Table S14 and Figure S10 in Additional file 1), sesame was estimated to have diverged from *U. gibba* approximately 98 million years ago (68.6 to 145.2 MYA), and from the tomato-potato lineage approximately 125 MYA (89.8 to 185.8 MYA) (Figure S11 in Additional file 1).

**Figure 2:**
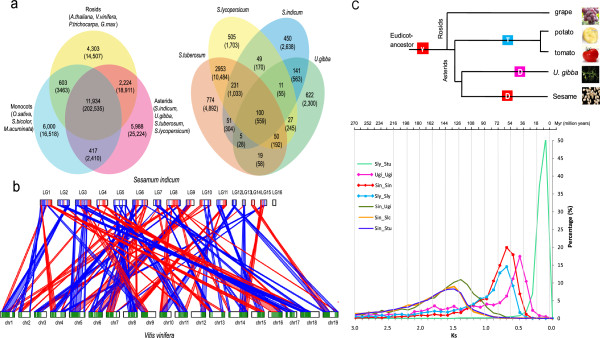
**Genome evolution of sesame. (a)** Left: shared and unique gene families in each lineage. Right: distribution of the asterids-specific gene families in sesame, *U. gibba*, tomato and potato. Gene numbers in related gene families are listed in brackets. **(b)** The relationships between grapevine and two subgenomes of sesame. These regions are listed in Table S15 in Additional file 1. For each segment in the grapevine chromosome, two obvious duplicated collinear segments from sesame were aligned. According to the retained gene ratio in duplicated segments, the high (red) and low-fractionated segments (blue) are represented. **(c)** Polyploidization events in the asterids I lineage. Age was estimated according to *K*s distribution. γ, gamma triplication event in eudicot ancestor; T, triplication event in the tomato-potato lineage; D, recent duplication event in sesame; Dmr, the most recent duplication event in *U. gibba*; Sin, *Sesamum indicum*; Sly, *Solanum lycopersicum*; Stu, *Solanum tuberosum*; Vvi, *Vitis vinifera*; Ugi, *Utricularia gibba*. See Supplementary Note 4 in Additional file 1 for more details.

We identified 11,934 shared dicot-monocot, 14,158 shared asterids-rosids (two clades of dicots) and 20,563 shared asterids lineage (sesame, *U. gibba*, tomato and potato) gene clusters (Figure 2a), representing their ancestral gene families. Moreover, we identified 450 gene families containing 2,638 genes, plus 3,972 single-copy genes that were specific to sesame (Table S14 and Figure S10 in Additional file 1; Data S3 and S4 in Additional file 2). Many of these genes were or encoded P450 genes, zinc finger proteins, transposases, transcription factors and disease resistance genes, suggesting their possible roles in species differentiation and adaptability in the sesame lineage or Lamiales.

Based on synonymous substitution rates (*K*_s_) and synteny relationships, we uncovered the dicot-specific gamma (γ) event and a novel independent whole genome duplication (WGD) event in the lineage leading to sesame (Figures S12 and S13 in Additional file 1). The recent WGD event in the sesame lineage was further corroborated in that the single grapevine region always aligned with two sesame segments (Figure 2b; Table S15 and Figure S14 in Additional file 1). We tentatively partitioned the recent sesame lineage-specific WGD genomic regions into two non-overlapping ‘subgenomes’. The two subgenomes of the WGD corresponded to approximately 61 Mb (7,781 genes) and approximately 74 Mb (7,975 genes) regions, respectively, constituting approximately 50% of the current sesame genome assembly. Among all referred grape genomic loci, 79.1% were found to have undergone substantial gene loss, with a copy retained in only one of duplicated syntenic regions (subgenomes) of the sesame genome (Supplementary Note and Tables S16 and S17 in Additional file 1; Data S5 and S6 in Additional file 2), following the WGD that occurred in the sesame lineage. To estimate the age of recent WGD events in the sesame genome, we extracted 1,239 duplicated sesame genes (both retained in two subgenomes; Data S7 in Additional file 2) and calculated their *K*_s_ values. We observed nearly parallel peaks and identical ranges in the *K*_s_ distributions of these duplicated gene pairs and those from the triplication event in tomato (Figure S15 in Additional file 1), which have been dated to approximately 71 (±19) MYA [26]. Therefore, the recent WGD event of sesame should have occurred independently in the parallel period of the triplication event in the tomato-potato lineage, but be older than the most recent duplication in *U. gibba* after their divergence (Figure 2c). Genes retained in duplicate are not evenly distributed among different functional categories [30], and an obvious bias was observed for the genes corresponding to transport, regulation, signal transduction and metabolism in sesame (Table S18 in Additional file 1). These over-retained genes may function necessarily in increasing the complexity of the regulatory networks accountable for the interaction between genotype and environment for the species after WGD.

#### Absence of amino-terminal Toll⁄interleukin-1 receptor nucleotide binding sites encoding resistance genes in sesame

Genes encoding nucleotide-binding sites (NBSs) are the largest class of plant disease resistance genes. Based on whether they contain a Toll⁄interleukin-1 receptor (TIR) domain [31], NBS resistance genes can be further categorized into two subclasses (TIR and non-TIR). We identified a total of 171 genes with an NBS domain in sesame (Table S19 in Additional file 1), and 65.2% were organized in tandem arrays (Figure S16 in Additional file 1). Intriguingly, all of the NBS-encoding resistance genes were not the TIR type. The absence of TIR domain-containing resistance genes has been reported generally in monocots [32], but is rare in eudicots, although it has been detected in sugar beet (*Beta vulgaris*) by PCR [33]. Analysis of homologous genes from 11 species, including 8 eudicots and 3 monocots, has also shown that sesame and monocots are absent from the OrthoMCL clusters of genes with the TIR domain (Data S8 in Additional file 2; Figure S17 in Additional file 1). The absence of NBS genes with the TIR domain in sesame was further examined by searching the gene-masked assembly and the unmapped reads; neither showed a TIR domain in sesame. Hence, the unambiguous absence of TIR domain-containing resistance genes at the whole genome scale in sesame, a species of eudicots, provides a new paradigm for the study of the evolution of resistance genes [34]. However, the mechanism that induced such loss and whether it is common in the order Lamiales require further elucidation.

### Quality characters of sesame

#### Molecular foundation for the high oil content of sesame

Elucidation of the sesame genome allowed the unprecedented opportunity to study oil biosynthesis to understand its high oil content. By searching the lipid-related gene database consisting of 222 functional families of *Arabidopsis thaliana*, we found that sesame has unexpectedly low gene copy numbers (708) compared to *A. thaliana* (736), soybean (1,298), grapevine (732), tomato (902) and rice (805) (Data S9 in Additional file 2). For the two edible oil crops, the conspicuous discrepancy between sesame and soybean in their oil content (approximately 55% versus approximately 20%, respectively, of dry seed) and their predicted lipid gene number (708 versus 1,298, respectively) implies that different properties or mechanisms of oil biosynthesis exist in the two distantly related oil crops.

**Figure 3:**
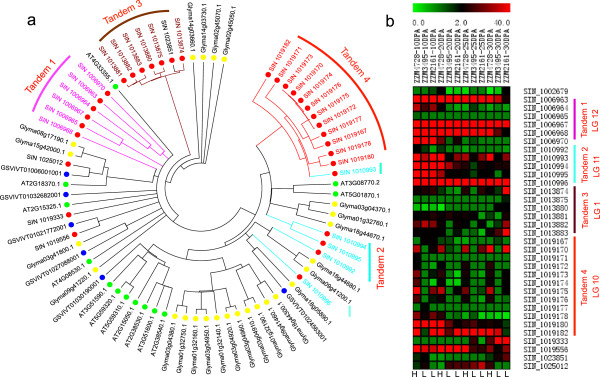
**Gene expansion of LTP1 in sesame. (a)** Maximum-likelihood tree showing expansions of LTP1 in sesame. Red dots, sesame; green dots, *Arabidopsis*; yellow dots, soybean; blue dots, grapevine. **(b)** Expression patterns and tandem arrays of LTP1 in sesame. Twelve transcriptomes corresponding to the seeds of 10, 20, 25 and 30 days post anthesis (DPA) of three accessions (from left to right): ZZM4728 (high oil content, 59% of seed), ZZM3495 (low oil content, 51% of seed) and ZZM2161 (low oil content, 48% of seed).

In contrast, soybean contains more copies than sesame of approximately 94.1% of the 222 lipid-related gene families. We found that the families encoding lipid transfer protein type 1 (LTP1), midchain alkane hydroxylase, FAD4-like desaturase (FAD4-like), and alcohol-forming fatty acyl-CoA reductase (AlcFAR) have been expanded by tandem duplication in sesame (Figures S18 to S20 in Additional file 1). Among these families, LTP1 is the largest, containing 34 genes with 29 clustering into 4 tandem arrays (Figure 3a). The high sequence similarity among the genes in tandem 3 and 4 suggested that each might have experienced a recent expansion. Of these LTP1 genes, more than 90% were expressed in a set of 12 sesame seed transcriptomes with RPKM (Reads per kilobase of exon per million reads mapped) greater than 1), confirming their functional activities in lipid biosynthesis (Figure 3b). Expansion and retention of these genes may reflect the selection for genomic variation corresponding to the production of high oil content during domestication because the enhancement of the LTP1 family may benefit oil accumulation by strengthening the transport of fatty acids, acyl-CoAs, and other lipid molecules [35]. In addition, the two cytosolic lipoxygenase (LOX) and lipid acyl hydrolase-like (LAH) families related to the degradation of lipids [36,37] are both contracted in sesame (8 LOXs and 18 LAHs) when compared to soybean (45 LOXs and 42 LAHs) (Figures S21 and S22 in Additional file 1). Based on these data, we speculate that the expansion of some lipid gene families, especially the type 1 lipid transport genes, and the contraction of lipid degradation-related families may lead to higher oil content in sesame than soybean.

#### Differentially expressed lipid genes in seed development

**Figure 4:**
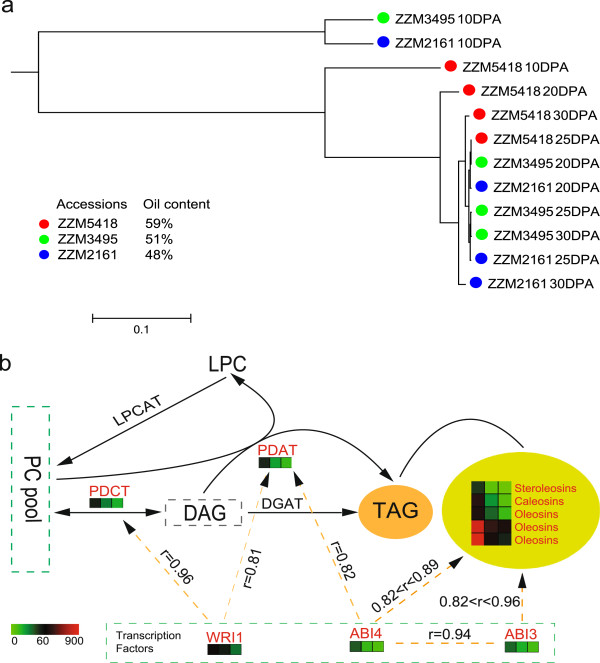
**Expression profiles of the lipid-related genes in sesame seed. (a)** Hierarchical clustering of the sesame seed samples based on the expression levels (RPKM) of 416 predicted lipid-related genes. **(b)** Differently expressed genes (DEGs) of 10 DPA seeds in the downstream part of the triacylglycerol (TAG) synthesis pathway. DEGs between the high and low lipid content accessions in this pathway are marked in red. Colored squares indicate the expression level (RPKM) of the 10 DEGs encoding phosphatidylcholine:diacylglycerol cholinephosphotransferase (PDCT), phospholipid: diacylglycerol acyltransferase (PDAT), oil-body proteins (steroleosin, caleosin and oleosin) and transcription factors (ABI3, ABI4 and WRI1) in the three accessions (from left to right): ZZM4728 (high oil content, 59% of seed), ZZM3495 (low oil content, 51% of seed), and ZZM2161 (low oil content, 48% of seed). The correlation (r) of expression patterns between transcription factors and genes was calculated using Pearson’s correlation coefficients (PCCs) based on all 12 transcriptomes. Dashed arrows indicate potential positive regulation.

To investigate the potential mechanism underlying the variation in oil content in sesame, we evaluated two accessions representing low oil content materials (48% and 51%) in germplasm, along with the high oil content cultivar ‘Zhongzhi No. 13’ (59%), each in four developmental stages (10, 20, 25, and 30 days post anthesis (DPA)) for RNA-Seq analysis (Table S1 in Additional file 1). Clustering of the expression profiles of the 416 sesame genes that were predicted to be orthologous to the lipid-related genes of *A. thaliana* (Supplementary Note in Additional file 1) clearly distinguished the 10 DPA from other stages and also the high and low oil sesame accessions (Figure 4a), suggesting that the determination of different oil content by lipid-related genes begins in the early stages of seed development.

After checking all oil-related pathways, we found that the genes expressed differentially at 10 DPA were enriched significantly in genes downstream of the triacylglycerol (TAG) biosynthesis pathway, including genes encoding phosphatidylcholine:diacylglycerol cholinephosphotransferase (PDCT) [38], phospholipid:diacylglycerol acyltransferase (PDAT) [39], oil-body proteins (steroleosin, caleosin and oleosin), and transcription factors (ABI3, ABI4 and WRI1) (Data S10 in Additional file 2). Until recently, the last step in TAG biosynthesis was assumed to be uniquely catalyzed by acylCoA:diacylglycerol acyltransferase (DGAT). However, some plants (for example, sunflower, castor bean and *Crepis palaestina*) and yeast were found to have an acyl-CoA-independent mechanism for TAG synthesis, which uses phospholipids as acyl donors and diacylglycerol as the acceptor [40,41]. In the present study, PDAT was expressed 2- to 3.5-fold higher in the one high-oil accession than the two low-oil accessions at 10 DPA (Figure 4b), whereas DGAT showed no significant differences in expression. These results are in accordance with the results in yeast, which revealed that overexpression of PDAT can increase TAG by two-fold in the early logarithmic phase [41,42]. Collectively, these data strongly suggested that the expression of PDAT in collaboration with other genes plays a pivotal role in shaping oil accumulation in the early stage of sesame seed development.

#### Population variation in sesame lipid-related genes

**Figure 5:**
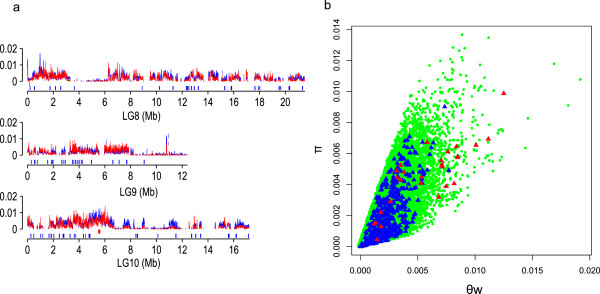
**Pairwise nucleotide diversity (*π*) and total polymorphism (*θw*) of sesame. (a)** Distributions of *π* (red) and *θw* (blue) of the sesame genome and positions of lipid-related genes (Figure S23 in Additional file 1). The two lines of bars below the axis of *π* or *θw* indicate the positions of the lipid-related genes in sesame. Blue bars, lipid-related genes except for LTP1; red bars, LTP1 genes. **(b)** Scatterplot of *π* and *θw* values of LTP1 genes (red triangles), non-LTP1 lipid-related genes (blue triangles) and the other genes (green circles).

To screen the sequence variation in lipid-related genes, 29 sesame accessions from 12 countries with oil content variation ranging from 48.6% to 59.8% were selected for genome resequencing. More than 120 Gb clean data corresponding to 13-fold genomic coverage for each accession were generated, resulting in the identification of 2,348,008 SNPs (Data S11 in Additional file 2). From these SNPs, population diversity (*π*) and Watterson’s estimator of segregating sites (*θw*) were estimated to be 0.0025 and 0.0032, respectively, in the population (Figure 5a; Figure S23 in Additional file 1). This genetic diversity is lower than that in rice [43], but higher than that in chickpea (*Cicer arietinum*) [44], watermelon (*Citrullus lanatus*) [45] and soybean [46] (Table S20 in Additional file 1). Lipid-related genes in sesame showed a wide variation with *π* values ranging from 0 to 0.0099, and were similar to the average of other genes (0.0021 versus 0.0020). In addition, lipid-related genes with high *π* values (top 10%) were significantly (*P* < 0.0001) enriched in the two biological processes of lipid transport and lipid localization. For example, most genes in the LTP1 family exhibited high diversity (Figure 5b). Specifically, the ‘youngest’ , in tandem 4, nearly had all of the highest *π* values in lipid genes. Furthermore, using read depth of coverage, we found that about a quarter of the 708 predicted lipid-related genes in sesame had copy number variations (Data S12 in Additional file 2) that were enriched significantly (*P* < 0.01) in the biological process of lipid transport and the molecular function of lipid binding. For the LTP1 genes, three of tandem 1, three of tandem 2, four of tandem 3 and eight of tandem 4 were observed to exhibit copy number variation (maximum of two to four copies) among the population (Data S13 in Additional file 2). The abundant variation in LTP1 might be associated with the intra-species differences in oil content.

We also found that the oleate desaturase (FAD2, *π* = 0.0016) and linoleate desaturase (FAD8, *π* = 0.0018) genes usually have low diversity, which may partially explain the low variation (40 ± 5% of oil) in oleic and linoleic acid content in sesame accessions [2]. For the oil-body protein families, higher variation was detected in the caleosin family (with an average *π* value of 0.0047) than in the oleosin (0.0025) and steroleosin (0.0024) families. In contrast, transcription regulatory families containing WRI1, ABI3 and ABI4 exhibited relatively low *π* values (<0.0008), indicating a conserved function for these transcription factors that participate in seed development and oil biosynthesis.

#### Genes for sesamin biosynthesis

Sesamin is an oil-soluble furofuran lignan typically present in sesame seed. It can increase oil stability and has been reported to be positively correlated with oil content [47]. Sesamin biosynthesis involves two key genes encoding dirigent protein (DIR) and piperitol/sesamin synthase (PSS), respectively [48] (Figure S24a in Additional file 1). By conducting a BLAST search against the 11 species (Table S14 in Additional file 1), we found that the DIR homologues are present in sesame (SIN_1015471) and tomato, but the PSSs are only detected in sesame (SIN_1025734), indicating the genetic foundation for the sesame-specific product.

The gene expression level usually correlates with its product [49]. In the four stages of three sesame accessions with different sesamin content (Table S1 in Additional file 1), DIR (SIN_1015471) expression was generally highest in the three 10 DPA seed samples, which decreased in the following stages. In contrast, PSS showed different patterns among the three accessions (Figure S24b in Additional file 1), with the highest expression levels detected at 20 DPA in the low sesamin accessions as reported previously [50] but at higher levels than detected at 10 DPA in the high sesamin accession. The higher PSS level in the early seed developmental stage in the high sesamin accession might be associated with its dual catalytic property, thereby producing more piperitol for subsequent sesamin biosynthesis. Although we expected to find PSS sequence variations between the high and low sesamin accessions, we found that it was especially conserved when we checked the 29 resequenced accessions (the three accessions used for RNA-Seq were also included). Thus, we speculated that other genes might regulate the different expressions of PSS; therefore, we selected a list of co-expressed genes of PSS for further study (Data S14 in Additional file 2). These genes are mainly involved in metabolic processes and catalytic activity (Figure S25 in Additional file 1).

## Conclusions

The *de novo* assembled genome of sesame offers a new whole genome sequence in the order Lamiales that follows the typical minute genome *U. gibba*. This information constitutes an important resource for genetic and evolutionary studies. The evolutionary scenario outlined for sesame clearly reveals a more recent WGD event at approximately 71 ± 19 MYA, which occurred after the split from tomato and potato and presents a new resource for studying the intricate paleopolyploidization processes in plants. The evolution of genes in Lamiales or asterids may be more complicated, considering the complete loss of the TIR-type NBS-encoding resistance genes in sesame, which undoubtedly presents a new paradigm in elucidating the fate of resistance genes along with their interactions with diseases. Moreover, determining whether similar mechanisms exist that induce or offset the loss of the TIR domain in both eudicots and monocots will be of great interest.

Although many studies have focused on the mechanisms of lipid biosynthesis and accumulation and the detection of lipid-related genes in different species, the genes involved in the dozens of complex oil biosynthesis pathways require further elucidation. For primary edible oil crops such as rapeseed, peanut and soybean, it is inextricable to encounter intertwined polyploidy or large genome sizes with many lipid-relates genes, which makes studies on oil biosynthesis much more daunting. In contrast, the higher oil content and fewer lipid-related genes in the small and diploid genome of sesame make it an invaluable potential model plant for studying oil biosynthesis. The *de novo* assembled genome, a set of 12 transcriptomes and 29 resequenced accessions provide a large resource for exploring the mechanisms underlying different oil content between sesame and soybean, as well as among sesame accessions. The extensive expansion and high diversity of LTP1 genes, and key genes differentially expressed in downstream of the TAG biosynthesis pathway should aid future genetic studies in sesame. Undoubtedly, future studies on sesame will help to improve the quantity and quality of edible oil crops in order to fight food and nutrition crises.

## Materials and methods

### DNA and RNA isolation

The genotype used for whole genome *de novo* sequencing was ‘Zhongzhi No. 13’, an elite sesame cultivar that has been introduced to most of the major sesame planting areas over the past 10 years. Genomic DNA was extracted from leaves with a standard CTAB (Cetyl trimethylammonium bromide) extraction method [51]. The materials used for RNA-Seq to analyze lipid and sesamin synthesis were three sesame accessions with different lipid and sesamin content (Table S1 in Additional file 1). The seeds of 10, 20, 25 and 30 DPA plants were sampled for RNA-Seq. The procedure described by Wei *et al*. [17] was used for RNA extraction and sequencing (or see Additional file 1).

### Whole genome shotgun sequencing and assembly

We carried out whole genome shotgun sequencing with the Illumina Hiseq 2000 platform. Eight paired-end sequencing libraries with insert sizes of approximately 180 bp, 500 bp, 800 bp, 2 kb, 5 kb, 10 kb and 20 kb were constructed, which generated a total data amount of 99.54 Gb. To reduce the effect of sequencing error on assembly, we applied a series of stringent filtering steps on read generation (see Supplementary Note in Additional file 1). After the above quality-control and filtering steps, 54.46 Gb of clean data, approximately 150-fold coverage of the predicted genome size, remained (Table S2 in Additional file 1; Data S1 in Additional file 2). The quality and quantity of the filtered data were checked by the distributions of the clean reads from every library (Figure S1 in Additional file 1). For all of the 37.63 Gb of clean data from short-insert size libraries, a custom program based on the *k-*mer frequency methodology was used to trim reads and correct bases [26]. Next, all of the remaining data were used for *de novo* genome assembly. We carried out the whole genome assembly using SOAPdenovo [30,52].

#### Contig construction

First, we split the reads from the short-insert size libraries into *k-*mers (k = 71) and constructed a *de Bruijn* graph. We then simplified the graph referring to the parameters, and lastly connected the *k-*mer path to produce the contig file.

#### Scaffold construction

All usable reads were realigned onto the contig sequences; the amount of shared paired end relationships between each pair of contigs and the rate of consistent and conflicting paired-ends were calculated to construct the scaffolds step by step, from short-insertion-size paired ends to long-insertion-size paired ends, and finally, scaffolds.

#### Gap filling

We used the tool GapCloser [53] to close the gaps inside the constructed scaffolds, which were mainly composed of repeats masked before scaffold construction. We used the paired-end information to retrieve the read pairs that had one end mapped to the unique contig and the other located in the gap region. Then, we preformed local assembly for these collected reads. Finally, about 274 Mb of the sesame genome was assembled, 98.8% of which is non-gapped sequences (Additional file 1).

### Estimation of genome size by flow cytometry

Flow cytometry was used to determine the DNA content of sesame [54]. Sesame samples and reference material were analyzed on an EPICS Elite ESP cytometer (Beckman-Coulter, Hialeah, FL, USA) with an air-cooled argon laser (Uniphase) at 488 nm using 20 mW. Salmon erythrocytes (2.16 pg/1C) were used as internal biological reference materials. Nuclear DNA content (in picograms) of sesame samples was estimated according to the following equation: 1C nuclear DNA content = (1C reference in picograms × Peak mean of sesame)/(Peak mean of reference). The number of base pairs per haploid genome was calculated based on the equivalent of 1 pg DNA = 978 Mb [55]. As a result, the C-value of sesame was estimated to be 0.34 pg/1C, and its genome size was estimated to be approximately 337 Mb (Figure S3 in Additional file 1).

### Anchoring of genome assembly to sesame genetic map

We used a combination method of specific length amplified fragment sequencing and experiment marker analysis to construct a new genetic map using 107 F_2_ lines derived from the Zhongzhi No.13/ZZM2289 population. In total, 2,719 SNPs, 97 insertions and deletions (indels) and 2,282 SSR markers were developed and screened against the population. After filtering the markers with low PCR quality, those having no polymorphism and those showing significantly distorted segregation in the population, the retained 45 indels, 259 SNPs and 124 SSR markers were used to construct the genetic map using JoinMap 3 software (Kyazma BV, Wageningen, Netherlands). Finally, we successfully constructed a genetic map that spans 1790.08 cM and has 406 markers, including 39 indels, 251 SNPs and 116 SSR markers. Based on the genetic map, 150 large scaffolds were anchored onto 16 pseudomolecules (see details in the Supplementary Note 3 in Additional file 1).

### Gene structure prediction and function annotation

To predict genes in the assembled genome, we used both homology-based and *de novo* methods. For the homology-based prediction, *A. thaliana*, grapevine, castor, and potato proteins were mapped onto the assembled genome using Genewise [56] to define gene models. For *de novo* prediction, Augustus [57] and GlimmerHMM were employed using appropriate parameters. Data from these complementary analyses were merged to produce a non-redundant reference gene set using GLEAN [58]. In addition, RNA-Seq data from multiple tissues (young roots, leaves, flowers, developing seeds, and shoot tips) from our previous study [17] were also incorporated to aid in gene annotation. RNA-Seq data were mapped to the assembled genome using TopHat [59], and transcriptome-based gene structures were obtained by cufflinks [60]. Then, we compared this gene set with the previous one to get the final non-redundant gene set of sesame (Tables S8 to S10 in Additional file 1). The non-coding gene predictions and gene function annotations were conducted as described in Supplementary Note 3 and Table S11 in Additional file 1.

### Repeat annotation

We identified repeat content in the sesame genome using a combination of *de novo* and homology-based approaches (Supplementary Note and Tables S12 and S13 in Additional file 1). Full-length LTR retrotransposons were identified by LTR_STRUC [61] and classified as *Gypsy*, *Copia* and other types of transposons using the program RepeatClassifer implemented in the RepeatModeler package [62]. Then the insertion time of LTR retrotransposons was dated according to the method described by JessyLabbé [63] (Supplementary Note and Figure S8 in Additional file 1).

### Evolution analysis

Gene clustering was conducted with OrthoMCL [64] by setting the main inflation value to 1.5 and other parameters as default. PHYML [65] was selected to reconstruct the phylogenetic tree based on the HKY85 model [66]. The program MCMCTree of the PAML package [67] was used to estimate species divergence time. Mcscan [68] was used to construct chromosome collinearity. Detailed descriptions about the identification of recent WGD events and two subgenomes are provided in Supplementary Note 4 in Additional file 1.

### Analysis of resistance genes in sesame

HMMER V3.0 [69] was used to screen the predicted sesame proteome against the raw hidden Markov model corresponding to the Pfam NBS (NB-ARC), and further build a sesame-specific NBS hidden Markov model for screening. The TIR and LRR domains were identified using local Pfam_Scan (-E 0.01 --domE 0.01). MARCOIL [70] with a threshold probability of 90 and the program paircoil2 [71] with a P-score cutoff of 0.025 were used as the settings for the CC motif identification.

The absence of NBS genes with a TIR domain in the sesame genome was further validated by checking the gene-masked assembly and the unassembled reads. For the masked assembly, we found nine NB-ARC fragments (>300 bp), but no TIR hit was obtained. Among all the unmapped reads, only 19 showed homology to the TIR domain, but all the reads together covered less than half of the TIR region. Considering the above results, NBS genes with a TIR domain were absent from sesame (for detailed methods see Supplementary Note 5 in Additional file 1).

### Analysis of important characteristics in the genome

The homologous lipid genes in sesame and other crops were identified by blastp (1e-5, identity >30%) based on the database of acyl-Lipid metabolism in *A. thaliana*[72] (for detailed methods see Supplementary Note 7 in Additional file 1).

### Genome resequencing and SNP calling

For each accession, a paired-end sequencing library with insert sizes of 500 bp was constructed and then sequenced on the Hiseq 2000 platform. The raw reads were then subjected to a series of stringent filtering steps that had been used in *de novo* genome assembly (Supplementary Note 1.3 in Additional file 1). Finally, we generated a total of more than 120 Gb clean data with each sample at over 13-fold sequence depth (Data S11 in Additional file 2). The clean reads were mapped to the assembled sesame genome using BWA software [73]. After mapping, SNPs were identified with read mapping quality ≥20 on the basis of the mpileup files generated by SAMtools [74] (Data S7 in Additional file 2). The SNPs extracted by the above process were first filtered by the sequencing depth: ≥30 and ≤581 using the vcfutils program in SAMtools. Then the raw SNP sites were further filtered using the following criteria: copy number ≤2 and a minimum of 5 bp apart with the exception of minor allele frequencies (≥0.05), whereby SNPs were retained when the distance between SNPs was less than 5 bp. Diversity parameters *π* and *θ*_*w*_ were measured using a window of 10 kb with a step of 1 kb [43,45].

Detection of copy number variations was performed as described by Zheng *et al.*[75] and Jiao *et al*. [76] (Supplementary Note 8 in Additional file 1).

### Data access

Genomic data generated by the whole project are available at NCBI under accession number APMJ00000000 [77]. WGS raw reads are deposited under the SRA study: SRA122008 [78]. The raw RNA-Seq data are deposited under the SRA study: SRA122023 [79]. Genome assembly, annotation and RNA-Seq data are also available at [80].

## Abbreviations

DPA: days post anthesis; indel: insertion and deletion; LTR: long terminal repeat; MYA: million years ago; NBS: nucleotide-binding site; PCR: polymerase chain reaction; SNP: single nucleotide polymorphism; SSR: simple sequence repeat; TAG: triacylglycerol; TIR: Toll⁄interleukin-1 receptor; WGD: whole genome duplication.

## Competing interests

The authors declare that they have no competing interests.

## Authors’ contributions

XRZ, JW and SYL contributed to the design of the research. LHW, SY, CS, RKV and CBT participated in the genome analysis and wrote the manuscript. YZZ, YL and WH participated in the study design and constructed the genetic map. LHW, SY, SYL and RKV participated in co-ordination and finalization of the manuscript. CS, XDZ, YW, DL, FL and CYX participated in the genome and transcriptome analyses. XLH and SST participated in the resequencing and analysis. YXZ and DHL prepared materials and performed the experiments. JYY performed the database construction. JYW, XX, YRL and SMH participated in the statistical analysis. All authors read and approved the final manuscript.

## Supplementary Material

Additional file 1 Supplementary Notes, Tables S1 to S20, and Figures S1 to S25. Click here for file

Additional file 2 Supplementary Data S1 to S14. Click here for file
