# Supplementary material for: Genome sequencing of the high oil crop sesame provides insight into oil biosynthesis
Source: Genome Biol. 2014 Feb 27;15(2):R39. doi: 10.1186/gb-2014-15-2-r39 (PMC4053841; doi:10.1186/gb-2014-15-2-r39)
Supplement: Additional file 1 — Supplementary Notes, Tables S1 to S20, and Figures S1 to S25. [file gb-2014-15-2-r39-S1.docx]

**Genome sequencing of the high oil crop sesame provides insight into oil biosynthesis**

Linhai Wang^1†^, Sheng Yu^2†^, Chaobo Tong^1†^, Yingzhong Zhao^1†^, Yan Liu^4†^, Chi Song^2^, Yanxin Zhang^1^, Xudong Zhang^2^, Ying Wang^2^, Wei Hua^1^, Donghua Li^1^, Dan Li^2^, Fang Li^2^, Jingyin Yu^1^, Chunyan Xu^2^, Xuelian Han^2^, Shunmou Huang^1^, Shuaishuai Tai^2^, Junyi Wang^2^, Xun Xu^2^, Yingrui Li^2^, Shengyi Liu^1*^, Rajeev K Varshney^5,6*^, Jun Wang^2,3*^ & Xiurong Zhang^1*^

^1^Oil Crops Research Institute of the Chinese Academy of Agricultural Sciences, Key Laboratory of Biology and Genetic Improvement of Oil Crops of the Ministry of Agriculture, Wuhan, 430062, China.

^2^Beijing Genomics Institute (BGI)-Shenzhen, Shenzhen, China.

^3^Department of Biology, University of Copenhagen, Copenhagen, Denmark.

^4^Yanzhuang oil CO., LTD, Hefei, 230038, China.

^5^International Crops Research Institute for the Semi-Arid Tropics (ICRISAT), Patancheru, India.

^6^CGIAR Generation Challenge Programme (GCP), c/o CIMMYT, Mexico DF, Mexico.

^†^These authors contributed equally to this work.

* Correspondence and requests of materials should be addressed to X.R.Z. ([zhangxr@oilcrops.cn](mailto:zhangxr@oilcrops.cn)), J.W. ([wangj@genomics.org.cn](mailto:wangj@genomics.org.cn)), R.K.V. ([R.K.Varshney@CGIAR.ORG](mailto:R.K.Varshney@CGIAR.ORG)) or S.Y.L.([liusy@oilcrops.cn](mailto:liusy@oilcrops.cn))

**Supplementary Information**

**Supplementary note**

**1. Genome sequencing and assembling**

1.1 Material preparation

1.2 Whole genome shotgun sequencing

1.3 Data filtering

1.4 Genome assembly

1.5 Estimate the sesame genome size by *k*-mer method

1.6 Estimate the genome size by Flow cytometry1.7 Check and screen contamination

1.8 Estimation of heterozygosity

1.9 Anchoring of genome assembly to sesame genetic map

**2. Assessment of genome assembly**

2.1 Assessing of the assembly with reads, ESTs and unigenes

2.2 Construction of 40 kb insert size fosmid library and sequencing

**3. Genome annotation**

3.1 Gene structure prediction

3.2 Gene function annotation

3.3 Non-coding genes prediction

3.4 Repeat annotation

**4. Evolution analysis**

4.1 The genome data used in evolution analysis

4.2 Gene clustering by OrthoMCL

4.3 Phylogeny construction and estimation of species divergence time

4.4 Synteny construction

4.5 Ancestral WGD event detection

**5. Identification of disease resistance genes**

**6. RNA-Seq for transcriptome analysis**

6.1 RNA extraction and library preparation

6.2 Data processing

**7. Analysis of lipid synthesis**

7.1 The potential sesame genes involved in lipid synthesis

7.2 Exploration of the mechanism underling the different lipid content in sesame seeds

**8. Genome resequencing**

8.1 SNP calling

8.2 Copy number variatiom (CNV) detection

**9. Analysis of sesamin synthesis in sesame**

**Supplementary Tables**

**Table S1**: The materials used for genome sequencing and RNA-Seq

**Table S2**: Data statistics of different insert size libraries used in genome assembly

**Table S3**: The assembly statistics of the sesame genome

**Table S4**: The genome assembly information of sesame and some other plants sequenced by next generation sequencing strategy

**Table S5**: Statistical information of the scaffolds anchored on each sesame linkage group

**Table S6**: Gene region coverage assessed by ESTs and unigenes

**Table S7**: Statistical results of the five sequenced fosmid clones aligned to the genome assembly with BLAT

**Table S8**: Gene prediction in the sesame genome

**Table S9**: Number of genes with protein or unigene support

**Table S10**: Comparison of the gene structure among asterid and rosid clades

**Table S11**: Noncoding genes in the sesame genome

**Table S12**: Repeat elements in the sesame genome

**Table S13**: Repeat elements in sesame, grape, potato and tomato genomes

**Table S14**: Gene families clustered by OrthoMCL in 11 species;

**Table S15**: The duplicated segments of sesame genome corresponding to all 19 grape chromosomes

**Table S16**: Gene retention in the two subgenomes of sesame

**Table S17:** The gene fractionation depth in the sesame genome

**Table S18**: Significantly enriched GO terms of duplicated genes from recent whole genome duplication (WGD) in the sesame genome

**Table S19**: Disease resistance proteins in sesame, potato, tomato and grape genomes

**Table S20**: Diversity levels of sesame and other species populations

**Supplementary Figures**

**Figure S1**: Distributions of the clean reads generated from the long-insert libraries

**Figure S2**: k-mer analysis to estimate the sesame genome size

**Figure S3**: Flow cytometric analysis of the genome size of sesame

**Figure S4**: Map of the sequence scaffolds along the sesame linkage groups (LGs)

**Figure S5**: Genetic distance vs. physical distance

**Figure S6**: The GC content distributions of sesame and other sequenced plants

**Figure S7**: Nucleotide alignments of five sequenced fosmids from sesame to their corresponding scaffold regions in the Illumina assembly

**Figure S8**: Distribution of the insertion time of long terminal repeats (LTRs) in sesame

**Figure S9**: Distribution of the divergence rates of LTRs

**Figure S10**: Gene number in each category defined by OrthoMCL

**Figure S11**: The phylogenetic relationship and split-time estimation based on all single-copy gene families shared by all species used

**Figure S12**: Distribution of the 4dTv distance between duplicated genes of syntenic regions in sesame (red bar) and tomato (green bar)

**Figure S13**: The Ks (synonymous) (x-axis) and Ka/Ks (y-axis) distribution for each syntenic block in the sesame genome

**Figure S14**: Two subgenomes originated from the ancestral WGD of the sesame genome were identified using the grape genome as reference

**Figure S15**: Distributions of the Ks and 4DTV of the duplicated genes in sesame and tomato

**Figure S16**: Distribution of nucleotide-binding site (NBS)-encoding resistance gene models along sesame linkage groups

**Figure S17:** Phylogenetic analysis of TIR-type NBS-encoding gene homologues belonging to the same OrthoMCL group generated from 10 species

**Figure S18**: Phylogenetic tree of the alcohol-forming fatty acyl-CoA reductase (AlcFAR) gene family

**Figure S19**: Phylogenetic tree of the FAD4-like desaturase (FAD4-like) gene family

**Figure S20**: Phylogenetic tree of the midchain alkane hydroxylase gene family

**Figure S21**: Phylogenetic tree of the lipoxygenase (LOX) gene family

**Figure S22**: Phylogenetic tree of the lipid acyl hydrolase-like (LAH) gene family

**Figure S23**: Distributions of π (red) and θw (blue) of the sesame genome and the positions of lipid-related genes

**Figure S24**: Expression patterns of the key genes involved in the sesamin biosynthesis pathway **Figure S25**: GO distribution of the genes correlated with (PCC > 0.9) PSS (SIN_1025734)

**Supplementary Note**

**1. Genome sequencing and assembling**

**1.1 Material preparation**

Sesame is generally taken as one of the self-pollinated plants regardless of insect-pollination. To guarantee the homozygosity of the genotype ‘Zhongzhi No. 13’, an elite sesame cultivar which has been introduced to most of the major sesame planting areas over the last 10 years, successive selfings were performed on the sample used for whole genome *de novo* sequencing, and then the genomic DNA was extracted from the etiolated leaves with a standard CTAB extraction method [[1](#_ENREF_1)].

The materials used to analyze oil and sesamin synthesis were ‘Zhongzhi No. 13’ and other two sesame accessions with different lipid and sesamin contents (Table S1 in Additional file 1). The seeds of 10, 20, 25 and 30 DPA (Days post anthesis) of each accession, i.e., twelve samples, were used for RNA-Seq and transcriptome analysis, respectively.

**1.2 Whole genome shotgun sequencing**

We carried out whole-genome shotgun sequencing with Illumina Hiseq 2000 platform. A total of 8 paired-end sequencing libraries with insert sizes of about 180 bp, 500 bp, 800bp, 2 kb, 5 kb, 10 kb and 20 kb were constructed and sequenced to obtain paired-end reads. In total, we generated 99.54 Gb data of paired-ends with a length of 100 bp and 50 bp in short (180 bp, 500 bp, 800 bp) and long (2 kb, 5 kb, 10 kb, 20 kb) insert size libraries, respectively. The sequencing depth was about 278.82🞨 when considering that the sesame genome size is 357 Mb by following *k*-mer method.

**1.3 Data filtering**

To reduce the effect of sequencing error to the assembly, we had taken a series of stringent filtering steps on reads generation. We filtered the following type of reads:

Type (1): Reads with ≥10% and ≥3% unidentified nucleotides for short and long insert size libraries, respectively.

Type (2): Reads from short-insert libraries having more than 40% bases with quality score less than 7, and reads from long-insert libraries that contained more than 20% bases with quality score less than 7.

Type (3): Reads with more than 10 bp aligned to the adapter sequence, allowing ≤ 2 bp mismatches.

Type (4): Small paired-end reads in short-insert libraries (except for paired-end reads from 180 bp insert library) that overlapped more than 10 bp with the corresponding paired end.

Type (5): Read1 and read2 of two paired-end reads that were completely identical (considered to be products of PCR duplication).

After the above quality control and filtering steps (Data S1 in Additional file 2), 54.46 Gb clean data, about 150🞨 of the predicted genome size was remained (Table S2 in Additional file 1). The data quality and quantity of the filtered long-insert libraries were checked by the distributions of the clean reads (Figure S1 in Additional file 1). For all of the 37.63 Gb clean data from short insert size libraries, a custom program SOAPec v2.01 (Correction tool for SOAPdenovo Version 2.01, <http://soap.genomics.org.cn>) was used for read trim and base correction. Then all the remained data was used for *de novo* genome assembly.

**1.4 Genome assembly**

We carried out the whole-genome assembly using SOAPdenovo [[2](#_ENREF_2), [3](#_ENREF_3)].

**Contig construction**: We firstly used all the reads from short-insert size libraries to construct de Bruijn graph with *k*-mer parameter –K71 –R, then simplified the graphs refers to the parameters by removing the tips and connections with low coverage, merging bubbles and masking small repeats, and lastly connected the *k*-mer path to get the contig file.

**Scaffold construction:** All the usable reads were realigned onto the contig sequences, and the amount of shared paired-end relationships between each pair of contigs, the rate of consistent and conflicting paired-ends, were calculated to construct the scaffolds step by step, from short-insert size paired-ends to long-insert paired-ends. To achieve higher accuracy, the parameter ‘pair_num_cutoff’ (the minimum required pairs of shared PE-reads to define a valid connection between each pair of contigs) in SOAPdenovo was increased from the default to 5, 5, 7 and 9 for 2kb, 5kb, 10kb and 20kb insert size data respectively, which generated the primary scaffolds spanning 277 Mb (≥ 200 bp), with 20 Mb or 7.2% of the total size were intra-scaffold gaps.

**Gap filling**: To close the gaps inside the constructed scaffolds, which were mainly composed of repeats that were masked before scaffold construction, the tool GapCloser (http://sourceforge.net/projects/soapdenovo2/files/GapCloser/) was used to fill the gaps based on the paired-end information of the read pairs that had one end mapped to the unique contig and the others located in the gap region. Finally, 93.6% of the intra-scaffold gaps, or 83.9% of the total gap length were filled, and about 274 Mb (≥ 200 bp) of sesame genome were assembled with 98.8% of which is non-gapped sequence.

The assembly consists of 26,239 contigs (≥ 200 bp) and 16,444 scaffolds (≥ 200 bp), with an N50 scaffold (N50 scaffold is a weighted median statistic indicating that 50% of the entire assembly is contained in scaffolds equal to or larger than this value) size of 2.1 Mb (Table S3 and S4 in Additional file 1). If only the scaffolds of ≥ 2 kb are considered, the genome assembly has 1,036 scaffolds. The GC ratio and distribution in whole genome level were measured with in-house perl scripts, and they are very close in sesame, tomato, potato and grape (Figure S6 in Additional file 1).

We also tried another tool, i.e. ABySS v1.3.6 to perform a second assembly [[4](#_ENREF_4)]. However, it resulted more fragmented contigs (N50, 14,102 bp) and scaffolds (N50, 432,640 bp), and shorter total length (249 Mb) than our current assembly, which indicated the present *denovo* assembly had reach to a relatively high extent.

**1.5 Estimate the sesame genome size by *k*-mer method**

Many studies had proved *k*-mer was proper to estimate the genome size [[5-7](#_ENREF_5)]. *k*-mer refers to a sequence with the length of k bp, and each unique *k*-mer within a genome dataset can be used to determine the discrete probability distributions of all possible *k*-mers and their frequency of occurrence. Genome size could be calculated using the total length of sequencing reads divided by sequencing depth. To estimate the sequencing depth of sesame genome, we counted the copy number of a certain *k*-mer (e.g., 17-mer) present in sequence reads, and plotted the distribution of copy numbers [[2](#_ENREF_2)]. The peak value of the frequency curve represents the overall sequencing depth. We used the algorithm: *N* × (*L* − *K* + 1)*/D* = *G*, where *N* is the total sequence read number, *L* is the average length of sequence reads and *K* is *k*-mer length, defined as 17 bp here. *G* denotes the genome size, and *D* is the overall depth estimated from *k*-mer distribution. Based on the method, the genome size of sesame was estimated to be 357 Mb (Figure S2 in Additional file 1).

**1.6 Estimate the genome size by Flow cytometry**

Flow cytometry (FCM) has become the method of choice to determine DNA content in plants, because of its convenient, fast and reliable [[8](#_ENREF_8)]. However, there were rare reports of the genome size of sesame measured by FCM. Herein, we estimated sesame genome size with the cultivar Zhongzhi No.13 by FCM. Voucher specimens were deposited in the National Medium-term Sesame Genebank of China, Oil Crops Research Institute, Chinese Academy of Agricultural Sciences, Wuhan, China. Salmon erythrocytes (2.16pg/1C) were used as internal biological reference materials.

The 5th – 8th leaves from shoot apex of each sesame sample and the biological references (30–50 mg) were finely chopped with a razor blade in 2.0 mL of cold MgSO_4_ extraction buffer containing 10mM MgSO_4_, 10mM KCl, 5mM 4-(2-Hydroxyethyl)-1-piperazineethanesulfonic acid (HEPES), 0.25%(w/v) Triton X-100 and 1.0%(w/v) polyvinylpyrrolidone (PVP) [[9](#_ENREF_9)]. After extraction, 50 µl of RNase and propidium iodide (PI) were added immediately prior to filtering through 42 µm nylon meshes [[9](#_ENREF_9), [10](#_ENREF_10)], then the extracts were kept on ice for further use.

Sesame sample and reference material were analyzed on an EPICS Elite ESP cytometer (Beckman-Coulter, Hialeah, Florida) with an air-cooled argon laser (Uniphase) at 488 nm, 20 mW. At least 2000 and generally 5000 nuclei were analyzed for each sample. Results are deduced from 1C nuclei in individuals considered diploid and are given as C-values. The nuclear DNA content (in pg) of sesame samples was estimated according to the equation: 1C nuclear DNA content = (1C reference in pg × peak means of sesame)/(peak mean of reference). The number of base pairs per haploid genome was calculated based on the equivalent of 1 pg DNA = 978 Mb [[11](#_ENREF_11)]. As a result, the C-value of sesame was estimated to be 0.34pg/1C, and its genome size was estimated about 337 Mb (Figure S3 in Additional file 1).

**1.7 Check and screen contamination**

Potential microbial contamination was checked by alignment against databases of bacterial and fungal genomes using Megablast (E-value < 1e-5, > 90% identity, > 200 bp length mapped to scaffold sequence). For checking the contamination of assembly with organelle DNA, sesame chloroplast DNA (153,324 bp, downloaded from http://www.ncbi.nlm.nih.gov/nuccore/378747301) and grape mitochondrion DNA (773,279bp, downloaded from <http://www.ncbi.nlm.nih.gov/>nuccore/224365609) were screened against the sesame genome assembly.

**1.8 Estimation of heterozygosity**

Heterozygosity of the sequenced genotype “Zhongzhi No. 13” was estimated according to the method mentioned in pigeonpea (*Cajanus cajan*) and bactrian camel [[12](#_ENREF_12), [13](#_ENREF_13)]. (i) All the high-quality reads of 180 bp (~52×) from the genomic DNA of “Zhongzhi No. 13” were mapped to the genome assembly using the software BWA [[14](#_ENREF_14)] with default parameters. (ii) The alignment was sorted and analyzed using SAMtools [[15](#_ENREF_15)] for SNP and InDels calling. The sites with sequencing depth of 5 to 105 and quality score greater than 20, were searched and retained as “effective sites”. (iii) Candidate SNPs and InDels in the “effective sites” were filtered using ‘vcfutils.pl varFilter’, and the heterozygous SNPs and InDels were then tallied up. (iv) Finally，the heterozygosity was estimated by the rate between the number of heterozygous sites (24,635 SNPs and 3,680 InDels) and effective sites (261,425,323 bp), resulting in the heterozygosity of “Zhongzhi No. 13” to be 1.08×10^-4^.

**1.9 Anchoring of genome assembly to sesame genetic map**

Up to the present project, there are no available sesame linkage maps with high quality and density to anchor the scaffolds onto chromosomes, so we constructed a new genetic map using the Zhongzhi No.13/ZZM2289 population, which consists of 107 F2 lines developed from a cross between Zhongzhi No.13 and ZZM2289 (from Oil Crops Research Institute, Chinese Academy of Agricultural Sciences). We used a combination method of SLAF (specific length amplified fragment) sequencing and experiment markers analysis to construct genetic map. We firstly detected 2,719 single nucleotide polymorphisms (SNPs) by SLAF-seq and constructed a new genetic map consisting of 257 markers (SNPs). However, it only anchored about 45% of estimated genome. We then compared the re–sequencing data of ZZM2289 to Zhongzhi No.13, and developed 97 insertion & deletion (InDel) markers to update the genetic map. Meanwhile, we screened the 200 top scaffolds that have less than 2 SNP or InDel markers for simple sequence repeat (SSR) loci, and designed 2,282 markers with each scaffold had more than 10. All the 2,282 SSR and 97 InDel markers were used to screen against the population. After filtering those markers with low PCR quality, those having no polymorphism and those showing significantly distorted segregation in the population, the retained 45 InDel and 124 SSR markers together with the 259 SNP makers were used to construct the genetic map using Joinmap3 software (http://www.kyazma.nl/index.php/mc.JoinMap). Finally, we successfully constructed a genetic map that spans 1,790.08 cM and has 406 markers including 39 InDel, 251 SNP and 116 SSR markers (Data S2 in Additional file 2).

Software E-PCR [[16](#_ENREF_16)] was used to map all makers onto the scaffold sequences of Zhongzhi No.13 by setting parameters: -d 100-500 -n1 -r + -O +. Only when the sequence of both primers perfectly and uniquely matched the scaffold sequence, it was considered to be anchored.

Based on the genetic map, 150 large scaffolds were arranged into 16 pseudomolecules (Table S5, and Figure S4 and S5 in Additional file 1), with 117 scaffolds oriented. In total, the 16 pseudomolecules harbor 85.3% of the assembly sequences in size and 91.7% of the predicted genes.

**2. Assessment of genome assembly**

**2.1 Assessing of the assembly with reads, ESTs and unigenes**

Different methods and data were employed to check the completeness of the assembly. We first mapped all the individual reads generated from the three short-insert libraries using BWA [[14](#_ENREF_14)] with default parameters. Overall, >94.7% of the reads could be mapped, and >85.5% of the reads could be mapped with proper insert size.

We downloaded all of the 3,328 reliable sesame ESTs [[17](#_ENREF_17)] that published in NCBI, and mapped them to the assembly genome with the BLAT software [[18](#_ENREF_18)] using default parameters. Analysis was done at different criteria of percent sequence homology and percent coverage by custom Perl scripts (Table S6 in Additional file 1). The results showed more than 99.3% of the ESTs were covered by the genome assembly. Furthermore, we mapped a set of multi-tissues (Young roots, leaves, flowers, developing seeds, and shoot tips) transcriptome assembly comprising 86,222 unigenes [[19](#_ENREF_19)] to the assembly genome with the BLAT as above, and found > 98.5% of the unigenes could be aligned to the genome assembly.

**2.2 Construction of 40 kb insert size fosmid library and sequencing**

The 40 kb insert size fosmid library was constructed according to the manual of the Copy Control Fosmid Library Production Kits (Epicentre Biotechnologies, USA). It was briefly operated as follows:

1. Purify DNA from the desired source (the kit does not supply materials for this step).

2. Shear the DNA to approximately 40-kb fragments.

3. End-repair the sheared DNA to blunt, 5'-phosphorylated ends.

4. Isolate the desired size range of end-repaired DNA by LMP agarose gel electrophoresis.

5. Purify the blunt-ended DNA from the LMP agarose gel.

6. Ligate the blunt-ended DNA to the Cloning-Ready CopyControl pCC1FOS or pCC2FOS Vector.

7. Package the ligated DNA and plate on EPI300-T1^R^plating cells. Grow clones overnight.

8. Pick CopyControl Fosmid clones of interest and induce them to high-copy number using the Copy-Control Fosmid Autoinduction Solution.

Finally, we constructed a 40 kb insert size fosmid library of more than 20,000 clones successfully. Then we selected 5 clones randomly to be sequenced thoroughly with ABI3730, and their size ranged from 33.5 to 38.6 kb (Table S7 in Additional file 1). We aligned the five sequences to the genome assembly with BLAT (default parameters), the results showed > 99.6% of these sequences were covered by the assembly (Figure S7 and Table S7 in Additional file 1).

**3. Genome annotation**

**3.1 Gene structure prediction**

To predict genes in the assembled genome, we used both homology-based and *de novo* methods. For the homology-based prediction, arabidopsis (*Arabidopsis thaliana*) [[20](#_ENREF_20)], grape (*Vitis vinifera*) [[21](#_ENREF_21)]*,* castor (*Ricinus communis*) [[22](#_ENREF_22)] and potato (*Solanum tuberosum*) [[23](#_ENREF_23)] proteins were mapped onto the assembled genome using Genewise [[24](#_ENREF_24)] to define gene models. For *de novo* prediction, Augustus [[25](#_ENREF_25)] and [Glimmer](#_ENREF_9)HMM [[26](#_ENREF_26)] were employed using appropriate parameters. Data from these complementary analyses were merged to produce a non-redundant reference gene set using GLEAN (http://sourceforge.net/projects/glean-gene/). In addition, RNA-Seq data of multi-tissues (Young roots, leaves, flowers, developing seeds, and shoot tips) from our previous study [[19](#_ENREF_19)] were also incorporated to aid gene annotation. Our RNA-seq data were mapped to the assembled genome using TopHat [[27](#_ENREF_27)], and transcriptome-based gene structures were obtained by cufflinks (<http://cufflinks.cbcb.umd.edu/>). Then, we compared this gene set with the previous gene set to get the final non-redundant gene set of sesame, and 27,148 genes were predicted with average transcript size of 3,171 bp (Table S8 and S10 in Additional file 1)*.* The mean length of coding sequence, exon, and intron of sesame are 1,180 bp, 249 bp and 439 bp, respectively (Table S10 in Additional file 1), and each gene has 4.7 exons in average.

**3.2 Gene function annotation**

Functions of sesame genes were assigned based on the best hit to proteins annotated in SwissProt and TrEMBL (Uniprot release 2011-01) databases using Blastp (E-value ≤ 1e-5). We annotated motifs and domains using InterProscan (Version 4.7) [[28](#_ENREF_28)] by searching against publicly available databases, including Pfam [[29](#_ENREF_29)], PRINTS[[30](#_ENREF_30)], PROSITE [[31](#_ENREF_31)], ProDom [[32](#_ENREF_32)] and SMART [[33](#_ENREF_33)]. Gene Ontology [[34](#_ENREF_34)] information was retrieved from InterPro. We also mapped the predicted sesame genes to KEGG [[35](#_ENREF_35)] pathways by searching KEGG databases (Release 58) and finding the best hit for each node (Table S9 in Additional file 1).

**3.3 Non-coding genes prediction**

Based on the assembled sesame genome, the tRNA genes were predicted by tRNAscan-SE-1.23 [[36](#_ENREF_36)] with eukaryote parameters. The rRNA fragments were identified by aligning the rRNA (5.8S, 18S rRNA and 28S) template sequences from plants (e.g., *Arabidopsis thaliana* and rice) using BlastN with E-value <1e-5. The miRNA and snRNA genes were predicted by INFERNAL software against the Rfam database (Release 9.1). All these information were listed in Table S11 in Additional file 1.

**3.4 Repeat annotation**

We identified repeat contents in sesame genome using a combination of *de novo* and homology-based approaches. First, we used three *de novo* software programs LTR_FINDER [[37](#_ENREF_37)] (Version 1.0.3), PILER [[38](#_ENREF_38)] and RepeatScout [[39](#_ENREF_39)] (Version 1.05) to build *de novo* consensus repeat database of sesame. Then we used RepeatMasker [[40](#_ENREF_40)] (Version 3.2.7) to identify repeats using the repeat database we had built. For homology-based identification, we used RepeatMasker and RepeatProteinMask (http://www.repeatmasker.org/, Version 3.2.2) to search the protein database in Repbase [[41](#_ENREF_41)] against the sesame genome to identify transposable elements. Then we combined the *de novo* prediction, the homolog prediction of repeat elements according to the coordination in the genome, and detected 77.9Mb repeat elements, about 28.5% of genome size in total (Table S12 and S13 in Additional file 1). We annotated the tandem repeats in the sesame genome using TRF [[42](#_ENREF_42)] (<http://tandem.bu.edu/trf/trf.html>, Version 4.04).

To infer the insertion time of LTR retrotransposon, full-length LTR retrotransposons were identified by LTR_STRUC [[43](#_ENREF_43)] with default parameters. The candidates from the LTR-STRUC search were classified as *Gypsy*, *Copia* and other types of transposons by the program RepeatClassifer implemented in the RepeatModeler package (<http://www.repeatmasker.org/RepeatModeler.html>). Then the left and right solo LTRs were aligned by MUSCLE [[44](#_ENREF_44)], and the distance between them was calculated by the Kimura two-parameter model using the distmat programme of EMBOSS package (<http://emboss.sourceforge.net/>). The insertion events of LTR retrotransposons were then dated by the method described by JessyLabbé [[45](#_ENREF_45)]. After ruling out low-complexity sequences, putative non-LTR retrotransposons and DNA transposons, 226 *Gypsy* and 295 *Copia* LTR retrotransposons were determined. The average insertion time of LTRs were estimated to 0.9 million years ago (MYA) with *Gypsy* 0.8 MYA and *Copia* 0.9 MYA, respectively (Figure S8 and S9 in Additional file 1).

**4. Evolution analysis**

**4.1 The genome data used in evolution analysis**

We downloaded the gene sets of 9 species from (1) Rosids clade of dicot plant: *A*. *thaliana* (TAIR10), *G*. *max* (JGI_7.0), *P*. *trichocarpa* (JGI_7.0), *V*. *vinifera* (Genoscope_12X); (2) Asterids clade of dicot plant: *S*. *tuberosum* (BGI), *S*. *lycopersicum* (ITAG2.3_release), *U. gibba* (CoGe V4.1); (3) Monocots: *S*. *bicolor* (JGI_7.0), *O*. *sativa* (IRGSP1.0), *M*. *acuminata* (http://banana-genome.cirad.fr/download.php) for following evolution analysis including gene clustering, phylogeny construction, divergence time estimation, and identification of chromosome collinearity *etc*. All the gene sets were dealt and filtered by following criteria:

1. Remove the gene whose length ≤150 bp and which of length has wrong triple.

2. Remove the gene which BLASTN against Repbase (E-value <1e-5, identity > 50% and coverage >80%).

3. Remove the gene which has internal stop codons in the CDS file.

4. Retain the gene which has longest alternative splicing sites.

5. If the gene has symbols for mix-bases, change the codon into NNN, corresponding proteins into X.

**4.2 Gene clustering by OrthoMCL**

Totally 359,180 genes from 11 whole genome sequenced species of plants were used for gene family clustering analysis. Firstly, blastp was used to generate the pairwise protein sequence with similarity of E-value less than 1e-5. Secondly, OrthoMCL [[46](#_ENREF_46)] was used to cluster similar genes by setting main inflation value 1.5 and other default parameters. Finally, 31,468 gene families containing 283,568 total genes from 11 species were generated. We identified 11,934 shared dicots–monocots, 14,158 shared asterids−rosids (two clades of dicots), and 20,563 shared asterids lineage (sesame, *Utricularia gibba*, tomato and potato) gene clusters (Figure 2a), representing their ancestral gene families, respectively. Moreover, we identified 450 gene families containing 2,638 genes, plus 3,972 single-copy genes, which were specific to sesame (Figure S10 in Additional file 1). The detailed statistics of clustering results were shown in Data S3 and S4 in Additional file 2, and Table S14 and Figure S10 in Additional file 1.

**4.3 Phylogeny construction and estimation of species divergence time**

From above OrthoMCL gene clusters, we extracted 490 clusters in which only one gene copy existed in each of above 11 species. Then we extracted 4-fold degenerate sites (4dTv) of all these orthologous single-copy genes in each species, and concatenated them to be one supergene for phylogeny construction. Software PHYML [[47](#_ENREF_47)] was selected to reconstruct the phylogenetic tree based on the HKY85 model [[48](#_ENREF_48)]. This tree was consistent with that deposited in NCBI, except for the *A. thaliana*-*P. trichocarpa*- *G. max* branch as that reported by Shulaev *et.al*.[[49](#_ENREF_49)]. The approximate likelihood-ratio (aLRT) [[50](#_ENREF_50)] for the branch *A. thaliana*-*P. trichocarpa* was 0.93, and over 0.98 for the others.

To validate the above phylogenetic tree, we also reconstructed 490 phylogenetic trees using the single copy gene families respectively. These gene trees were further subjected to inferring the species tree by the software DupTree [[51](#_ENREF_51)], which showed the new constructed species tree consistently matched the supergene tree. Thus, the supergene phylogenetic tree was reliable.

We further estimated the divergence time for 10 species based on all single-copy orthologous genes and 4-fold degenerate sites. Markov chain Monte Carlo algorithm for Bayes estimation was adopted to estimate the neutral evolutionary rate and species divergence time using the program MCMCTree of the PAML package [[52](#_ENREF_52)], by setting two fixed corrected time points: ~7.3 (7.2-7.4) Million years (Myr) split time between potato and tomato [[53](#_ENREF_53)], 173.2 (129.1-239.8) Myr split time between dicots and monocots [[21](#_ENREF_21)]. The phylogenetic relationship among these species and the split time estimation between species were shown on Figure S11 in Additional file 1. The sesame was placed in the asterids lineages and estimated to split from tomato-potato ~125 million years ago (89.8 - 185.8 MYA).

**4.4 Synteny construction**

MCscan (<http://chibba.agtec.uga.edu/duplication/mcscan>) was used to construct the chromosome collinearity within sesame and tomato, respectively. Syntenic blocks containing at least 6 genes were obtained based on the similarity gene pairs (blastp: E<1e-5). We extracted all the duplicated gene pairs (sesame: 6,204, tomato: 4,265) from syntenic blocks in the two species to further calculate the 4dTv distances using the HKY substitution model [[48](#_ENREF_48)]. The distribution of 4dTv (Figure S12 in Additional file 1) confirmed the ancient gamma triplication event and recent reported WGT (whole genome triplication) event (~71±19 Myr) in tomato-potato lineage [[53](#_ENREF_53)]. For sesame, it shared the ancient pan-dicots gamma event with tomato, from which duplicated genes in sesame and tomato diverged in 4dTv of ~0.75. More importantly, a more recent sesame-lineage specific whole genome duplication event (see below) have occurred (corresponds to 4dTv peak ~0.27) after its split from tomato-potato ancestor.

We also calculated the average synonymous (*K*_s_) and non-synonymous (*K*_a_) substitution rates of all 6,204 duplicated gene pairs in each paired syntenic block within sesame itself (Figure S13 in Additional file 1). Obviously, two groups of syntenic block could be divided by *K*_s_ distribution: One group corresponds to gamma WGT event and distributed in *K*_s_ range of 1.5 - 2.5 and another group corresponds to 0.5 − 1 *K*_s_ value from a more recent WGD event.

**4.5** **Ancestral WGD event detection**

Considering the grape genome have only owned one ancestral pan-eudicot shared whole genome triplication event (known as “γ” event) and no other WGD (whole genome duplication) events occurred during the subsequent evolution [[21](#_ENREF_21)], it was especially suitable as a reference to detect the WGD event in other plants [[53](#_ENREF_53)] since it kept comparative completed ancestral chromosomal structure. The main procedures for detection of duplicated segments originated from WGD are as follows:

**Step1**: We downloaded grape gene dataset (totally 26,346 gene models) from Genoscope website ([www.genoscope.cns.fr/externe/Download/Projets](http://www.genoscope.cns.fr/externe/Download/Projets)), and used it as references. Blastp were used to construct grape-sesame gene pairs (E-value threshold 1e-5). Finally, sesame-grape gene pairs containing 21,638 sesame genes and 12,478 grape genes were generated.

**Step2**: Software Mcscan (<http://chibba.agtec.uga.edu/duplication/mcscan>) was used to generate the syntenic relationship between sesame and grape chromosomes based on the gene pairs from step1. We set 15 genes as the minimal number of genes required to call synteny and other default parameters. Finally, 182 sesame-grape syntenic blocks containing 8,200 sesame-grape orthologous gene pairs were obtained.

**Step3**: We observed that there are always two sesame genome segments can be aligned to single grape genome segments. We further examined these duplicated segments carefully, and filter some low-scored and short collinear segments that shows to be great fractionated, and also with overlap with other high-quality segments. Finally, the two non-overlapping subgenomes of sesame genome were isolated and visualized in Figure S14 and Table S15 in Additional file 1.

The two subgenomes of the whole genome duplication correspond to ~61Mb (7,781 genes) and ~74Mb (7,975 genes) regions, respectively (Figure S14 in Additional file 1), constituting approximately 50% of the current sesame genome assembly. Within the two subgenomes, 1,239 presumed ancestor loci have been retained in both corresponding location after WGD (Data S7 in Additional file 2).

These 1,239 duplicated gene pairs were used to calculate the average synonymous (*K*_s_) for dating the WGD event. Additionally, we downloaded the duplicated genes derived from tomato-potato lineage specific WGT event for *K*_s_ calculation and time estimation.

***K*s distribution analysis (Figure 2c):** We used the average synonymous substitutions (*K*s) from different events for time estimation: 1) 1,239 duplicated gene pairs derived from and represented sesame-lineage specific WGD event; 2) 1,692 duplicated gene pairs derived from and represented tomato-potato lineage specific WGT event [[53](#_ENREF_53)] (Supplementary Table 61 in tomato genome paper); 3) 2,415 duplicated gene pairs derived from and represented *U. gibba*. 4) 18,957 orthologous gene pairs between potato and tomato were obtained from reciprocal best hit of BLAST, and represented the split and divergence between them; 5) 12,903 orthologous gene pairs between sesame and tomato were obtained from reciprocal best hit of BLAST, and represented the split and divergence between them; 6) 11,991 orthologous gene pairs between sesame and potato were obtained from reciprocal best hit of BLAST, and represented the split and divergence between them. 7) 10,827 orthologous gene pairs between sesame and *U. gibba* were obtained from reciprocal best hit of BLAST, and represented the split and divergence between them. All these *K*s distribution curves from these events are shown in **Figure 2c**.

**Fractionation depth analysis**: We investigated the gene loss/retention in the duplicated syntenic regions (subgenomes) derived from the recent WGD event in sesame in two ways. First, we found 79.1% of the genes in the two duplicated regions (subgenomes) of sesame syntenic to grape genomic loci have only one copy retained (Table S16 in additional file 1, Data S5 in additional file 2), indicating substantial gene loss following the WGD occurred in sesame-lineage. Second, for further conducting fractionation depth of duplicated syntenic regions derived from all polyploidization events containing the recent WGD and the old gamma (γ) events, we tested a series of gradually loose parameters for construction of grape-sesame (1: n) syntenic blocks in consideration of the high degree of fractionation of gamma (γ)-derived segments due to long evolutionary time and repeated fractionation affected by the following recent WGD in sesame (Table S17 in additional file 1, Data S6 in additional file 2 ). The fractionation depth of grape-sesame (1:1) was ~75% although the recent WGD and old gamma (γ) event were considered for each sesame genomic locus at the same time. The above results both indicated that substantial gene loss following whole genome duplication had occurred and reasonably were responsible for the low gene count in sesame.

**5. Identification of disease resistance genes**

The predicted proteome of sesame was firstly searched against all Pfam-A families (release 26.0, downloaded from [ftp://ftp.sanger.ac.uk/pub/databases/Pfa​m](ftp://ftp.sanger.ac.uk/pub/databases/Pfam)) using the “pfam_scan” perl script (version 1.3) downloaded from the Pfam website. Default thresholds were used, which were hand-curated for every family and designed to minimise false positives. Those containing NB-ARC (PF00931) domains were regarded as disease resistance genes, and TIR (PF01582) and LRR (PF00560, PF07723, PF07725, PF12799, PF13306, PF13516, PF13504, PF13855, and PF14580) domains were assigned to them then. As for the CC motif in the N-terminal region, all the disease resistance genes were searched using the program paircoil2 [[54](#_ENREF_54)] with a P-score cut-off of 0.025 (Table S19 and Figure S16 in Additional file 1). Finally, the predicted disease resistance genes were subjected to manually classification according to the domains they contained. TIR domains’ absence in disease resistance genes in sesame was further confirmed by ‘hmmsearch’ programa in HMMER V3.0 (<http://hmmer.janelia.org/>) using -E and -domE cutoff as high as 1.

The absence of the NBS gene with a TIR domain in the sesame genome was further validated by checking the gene-masked assembly and the unassembled reads. First, a DNA HMM-profile of the TIR domain was built using the hmmbuild programme in HMMER (http://hmmer.janelia.org/software) based on the 16 well-studied TIR-NBS genes selected manually based on the ‘Domain organisation’ information in Pfam (http://pfam.sanger.ac.uk/). Second, the predicted protein-coding regions of the assembly were masked and subjected to the home-build DNA HMM-profile using the nhmmer programme for homologous regions. Then, all the unmapped reads were searched against the DNA HMM-profile using nhmmer.

For the masked assembly, we found 9 NB-ARC fragments (> 300 bp), but no TIR hit was obtained. Among all the unmapped reads, only 19 showed homology to TIR domain, but all the reads together covered less than half of the TIR region. Considering the above results, the NBS genes with a TIR domain were absent from sesame

**6. RNA-Seq for transcriptome analysis**

**6. 1 RNA extraction and library preparation**

RNA extraction and sequencing used the same procedure refers to Wei *et al*. [[19](#_ENREF_19)]. Briefly, total RNA of every sample was isolated using the TRIzol reagent according to the manufacturer’s instructions (Invitrogen). The total RNA concentration was quantified using an ultraviolet (UV) spectrophotometer, and RNA quality was assessed on 1.0% denaturing agarose gels. The qualified RNA was treated with DNase I prior to library construction, and Magnetic Oligo (dT) Beads was used to purified the poly-(A) mRNA. Then the mRNA was fragmented by treatment with divalent cations and heat. The cleaved RNA fragments were transcribed into first strand cDNA using reverse transcriptase and random hexamer-primers, followed by second-strand cDNA synthesis using DNA polymerase I and RNaseH. The double-stranded cDNA was further subjected to end repair using T4 DNA polymerase, the Klenow fragment, and T4 polynucleotide kinase followed by a single <A> base addition using Klenow 3’ to 5’ exo-polymerase, then ligated with an adapter or index adapter using T4 DNA ligase. Adaptor-ligated fragments were separated by size on an agarose gel, and the desired range of cDNA fragments (200 ± 25 bp) were excised from the gel. PCR was performed to selectively enrich and amplify the cDNA fragments. After validation with an Agilent 2100 Bioanalyzer and ABI StepOnePlus RealTime PCR System, the cDNA library was sequenced on a flow cell using an Illumina HiSeq2000 sequencing platform.

**6.2 Data processing**

The raw reads were cleaned by removing reads with adapters and unknown bases (>5%), and low quality reads (the percentage of low quality bases is over 30% in a read, we define the low quality base to be the base whose sequencing quality is no more than 20). After filtering, the remaining reads are called "clean reads" and used for downstream bioinformatics analysis. Clean reads are mapped to a reference genome using SOAPaligner/SOAP2 [[2](#_ENREF_2), [3](#_ENREF_3)]. No more than 3 mismatches are allowed in the alignment.

**7. Analysis of lipid synthesis**

**7.1 The potential sesame genes involved in lipid synthesis**

The 736 genes of *A.thaliana* involved in Acyl-Lipid Metabolism were downloaded from <http://aralip.plantbiology.msu.edu>, and they were sorted by cellular function and gene families. Using blastp (E-value < 1e-5, identity > 30%), the homologous gene in sesame and other 4 crops (*V. vinifera*, *G*. *max*, *O. sativa*, *S.lycopersicum*) were identified for number comparison. The gene numbers were listed in Data S9 in Additional file 2.

**7.2 Exploration of the mechanism underlying the different lipid content in sesame seeds**

When analyzing the mechanism underlying the different lipid contents in sesame seeds, we had planned to use the orthologous lipid-related genes of sesame to A.thaliana. We firstly predicted 425 orthologs using the frequent method of Reciprocal Best blast Hit (RBH) [[55](#_ENREF_55), [56](#_ENREF_56)]. Then, we checked the syntenic relationships of these predicted orthologous genes, but found only half (220) of them locate in the syntenic blocks between sesame and A.thaliana, which may due to the distant divergence between them. Next, we check the Pfam containing both the predicted orthologs in the two species, and filtered out 20 sesame genes that have no coincident domain to A.thaliana. However, 11 of the 20 genes were included in the 220 syntenic relationships. Collectively, we predicted 416 orthologous lipid-related genes in sesame to A.thaliana. According to the expression level (RPKM) of these genes, hierarchical clustering based on Spearman correlational distance of the seed samples of ‘zhongzhi No. 13’ (ZZM4728), ZZM2161 and ZZM3495 was conducted with MeV[[57](#_ENREF_57)], then viewed in MEGA [[58](#_ENREF_58)]. Genes were sorted to pathway according to <http://aralip.plantbiology.msu.edu/downloads>. Thirty-two genes were identified as different expressed genes (DEGs) between ZZM4728 and ZZM3495 in 10 DPA, and forty-nine genes between ZZM4728 and ZZM2161. Pathway enrichment analysis of the DEGs in 10DPA was conducted with enrichment pipeline [[59](#_ENREF_59)] using the 425 orthologous genes as background. The correlation of expression pattern between transcription factors and other DEGs were calculated with Pearson's correlation coefficients (PCC) based on the twelve transcriptomes of the three accessions.

**8. Genome resequencing**

We selected 29 sesame accessions for genome resequencing, including sixteen from China and thirteen from America, Afghanistan, Egypt, Guinea, India, Korea, Myanmar, Mozambique, Philippine, United Arab Emirates, Viet Nam, respectively. For each accession, a paired-end sequencing library with insert size of 500 bp was constructed and then sequenced on the HiSeq 2000 platform. The raw reads were then subjected to a series of stringent filtering steps that had been used in *denovo* genome assembly (see supplementary note 1.2). Finally, we generated more than 120 Gb clean data totally with each sample at over 13-fold sequence depth (Data S11 in Additional file 2).

**8.1 SNP calling**

These reads were mapped to the assembled sesame genome of “Zhongzhi No.13” using BWA software [[14](#_ENREF_14)]. The detailed parameters used were as follows:

“bwa aln -m 200000 -o 1 -e 30 -i 15 -l 35-L -I -t 4 -n 0.04 -R 20 –f”

“bwa sampe -a 800”

Considering all the accessions as a group,“mpileup”pileSAMtools [[15](#_ENREF_15)] was used to detect the raw population SNP dataset by reads with the mapping quality ≥ 20. The detailed parameters were as follows:

“samtools mpileup -uf -b -D| bcftools view -bvcgI -p 0.99 “

Using the program 9vcfutils”cfutSAMtools, SNPs extracted by above process were first filtered by the sequencing depth: ≥ 30 and ≤ 581. The detailed parameters used were as follows:

“perl vcfutils.pl varFilter -d 30 -D 581”

Raw SNP sites were further filtered on the following criteria: copy number ≤ 2, a minimum of 5 bp apart with the exception of minor allele frequencies (MAF ≥ 0.05) where SNPs were retained when the distance between SNPs was less than 5 bp. The diversity parameters *π* and *θ_w_* were measured using a window of 10 kb with a sliding window of 1 kb [[60](#_ENREF_60), [61](#_ENREF_61)].

**8.2 Copy number variatiom (CNV) detection**

The method to detect CNV refers to Zhang *et al.* and Jiao *et al*. [[62](#_ENREF_62), [63](#_ENREF_63)]. Firstly, read depth of every 100-bp window was computed by counting the start position of reads within this window. Considering the bias in read depth caused by GC content, we first adjusted the read depth of every window with the equation Adjusted_read Depth = readDepth × m/ (m_GC_), where Adjusted_read Depth is the adjusted read depth, readDepth is the read depth of the window, m is the median value of all windows of a chromosome and m_GC_ is the median read depth of all windows that have the same GC content as the adjusted window. After adjustment, the DNA sequences were separated into fragments according to the depth of each base gotten from the alignment results. Sequently, we calculated the P value for each fragment to estimate its probability to be a CNV. The *P*-value was calculated as the probability of each observed depth (d) under the distribution of a simulated Poisson distributed data set whose expected value (E(d)) equals the observed mean depth. If d < E(d), the *P*-value = P(x, the d)) equa*P*-value = P (x the d)) equals the observed mean depth.ribution of *P*-value becomes smaller. Finally, fragments that passed the criteria (fragment length longer than 2 kb, *P*-valued the criteria (fragment length longer than 2 kb, were kept as CNVs.

**9. Analysis of sesamin synthesis in sesame**

Homologous genes of dirigent protein (DIR) and piperitol/sesamin synthase (PSS) [[64](#_ENREF_64)] were detected by alignment DIR (GenBank accessions AY560651) and PSS genes (CYP81Q1, GenBank accessions AB194714) to the sesame predicted genes using blastp, respectively. PCC (Pearson’s correlation coefficients) value of a pair of gene expression pattern, considering sample redundancy, was calculated following the formula of the online help page (<http://atted.jp/help/coex_cal.shtml>) (Data S14 in Additional file 2, and Figure S25 in Additional file 1).

**Supplementary Tables**

**Table S1** The materials used for genome sequencing and RNA-Seq

| **Material** | **Lipid**  **(g/100 g seed)** | **Sesamin**  **(g/100 g seed)** | **Sesamolin**  **(g/100 g seed)** | **Utility** |
| --- | --- | --- | --- | --- |
| **Zhongzhi No.13**  **(ZZM4728)** | 59.1 | 0.48 | 0.28 | Genome sequencing and RNA-Seq |
| **ZZM2161** | 48.4 | 0.13 | 0.26 | RNA-Seq |
| **ZZM3495** | 50.95 | 1.11 | 0.70 | RNA-Seq |

Data sets of samples from RNA-Seq:

| **Material** | 10 DPA (Gb) | 20 DPA (Gb) | 25 DPA (Gb) | 30 DPA (Gb) |
| --- | --- | --- | --- | --- |
| **ZZM4728** | 2.13 | 2.21 | 2.27 | 2.26 |
| **ZZM2161** | 2.14 | 2.28 | 2.28 | 2.21 |
| **ZZM3495 2223** | 2.34 | 2.25 | 2.28 | 2.29 |

DPA: Days post anthesis.

**Table S2** Data statistics of different insert size libraries used in genome assembly

| **Pair-end libraries** | **Insert size** (mean/SD) | **Average reads length(bp)** | **Total data(Gb)** | **Sequence**  **depth (🞨)** |
| --- | --- | --- | --- | --- |
| **Filtered Reads** | 180bp (154/9) | 95 | 18.51 | 51.84 |
|  | 500bp (518/64) | 95 | 9.13 | 25.58 |
|  | 800bp (749/25) | 85 | 9.99 | 27.98 |
|  | 2kb (2,355/177) | 49 | 8.26 | 23.15 |
|  | 5kb (5,325/394) | 49 | 4.46 | 12.50 |
|  | 10kb (10,807/1,341) | 49 | 1.99 | 5.57 |
|  | 20kb^a^ (17,367/3,881, 19,492/5,171) | 49 | 2.11 | 5.91 |
| **Total** | / | / | 54.46 | 152.54 |

^a^ two libraries were constructed..

Note: DNA libraries with different insert sizes were constructed and sequenced. In total, 99.54 Gb raw data were generated and the sequencing depth is about 278.82🞨. After data filtering, more than 150🞨 clean data were used in the genome assembly.

**Table S3** The assembly statistics of the sesame genome

|  | **Contig** | | **Scaffold** | |
| --- | --- | --- | --- | --- |
|  | **Size(bp)** | **Number** | **Size(bp)** | **Number** |
| **N90** | 11,433 | 5,534 | 268,228 | 169 |
| **N80** | 21,955 | 3,886 | 689,815 | 110 |
| **N70** | 31,432 | 2,864 | 1,079,037 | 77 |
| **N60** | 41,644 | 2,125 | 1,623,838 | 57 |
| **N50** | 52,169 | 1,545 | 2,096,681 | 42 |
| **Longest** | 471,223 | / | 6,995,259 | / |
| **Total Size** | 270,364,434 | / | 273,596,034 | / |
| **Total Number(**≥ **200 bp)** | / | 26,239 | / | 16,444 |
| **Total Number(**≥ **2 kb)** | / | 9,023 | / | 1,036 |
| **Length of Ns** | / | / | 3,231,600 |  |

**Table S4** The genome assembly information of sesame and some other plants sequenced by next generation sequencing strategy

| **Iterm** | ***S*. *indicum*** | ***C*. *sativus*** | ***S*. *italica*** | ***C*. *cajan*** | ***B*. *rapa*** |
| --- | --- | --- | --- | --- | --- |
| **Predicted genome size(Mb)** | 357 | 367 | 490 | 833 | 485 |
| **Sequence data (Gb)** | 99.5 | 26.5 | / | 237.2 | 36 |
| **Clean data(Gb)** | 54.5 | / | 40 | 130.7 | / |
| **Depth based on raw data** | 278.7 | 72.2 | / | 284.8 | 72 |
| **Depth based on clean data** | 152.7 | / | 81.6 | 163.4 | / |
| **N50 contig (kb)** | 52 | 12.5 | 25.4 | 21.95 | 27 |
| **N50 scaffold (kb)** | 2,097 | 172 | 1,000 | 516 | 1,971 |
| **Percent of assembly** | 77.4% | 70.0% | 86.0% | 72.7% | 58.5% |
| **Predicted gene** | 27,148 | 26,682 | 38,801 | 48,680 | 41,174 |
| **Percent of repeat** | 28.5% | 24.0% | 46.0% | 51.7% | 39.5% |

“/“ indicates no available information from publication.

**Table S5** Statistical information of the scaffolds anchored on each sesame linkage group

| **Linkage**  **group** | **Number of**  **markers** | **Number**  **of scaffolds**  **(all)** | **Number**  **of scaffolds**  **(oriented)** | **Total length**  **(bp, with NNs)** | **Total length**  **(bp, without NNs)** |
| --- | --- | --- | --- | --- | --- |
| **LG1** | 32 | 10 | 9 | 18,577,331 | 18,353,930 |
| **LG2** | 26 | 8 | 7 | 18,500,646 | 18,309,402 |
| **LG3** | 48 | 14 | 12 | 24,928,530 | 24,586,084 |
| **LG4** | 43 | 18 | 10 | 17,356,267 | 16,975,142 |
| **LG5** | 33 | 13 | 9 | 18,898,134 | 18,612,917 |
| **LG6** | 36 | 13 | 12 | 25,289,714 | 25,012,497 |
| **LG7** | 30 | 14 | 10 | 11,725,536 | 11,519,752 |
| **LG8** | 27 | 9 | 8 | 21,523,998 | 21,308,197 |
| **LG9** | 14 | 6 | 6 | 12,411,895 | 12,246,513 |
| **LG10** | 24 | 10 | 7 | 17,245,970 | 17,055,383 |
| **LG11** | 27 | 9 | 7 | 15,446,199 | 15,265,867 |
| **LG12** | 19 | 6 | 6 | 6,373,461 | 6,278,374 |
| **LG13** | 17 | 7 | 6 | 5,050,363 | 4,947,375 |
| **LG14** | 6 | 4 | 2 | 4,882,680 | 4,824,773 |
| **LG15** | 14 | 5 | 4 | 10,047,770 | 9,943,669 |
| **LG16** | 7 | 4 | 2 | 4,963,887 | 4,883,938 |
| **Total** | 403 | 150 | 117 | 233,222,381 | 230,123,813 |

**Table S6** Gene region coverage assessed by ESTs and unigenes. The unigenes were assembled by RNA sequencing data and aligned to the genome assembly. The proportion of ESTs or unigenes aligned to the genome assembly was used to represent the gene region coverage.

| **EST** | | | | | | | | | |
| --- | --- | --- | --- | --- | --- | --- | --- | --- | --- |
| Dataset | Number | | Total  length (bp) | Covered by assembly (%) | With >90% Sequence in one Scaffold | | With >50% Sequence in one Scaffold | | |
|  |  |  |  |  | Number | Percentage (%) | Number | | Percentage (%) |
| All | 3,328 | | 1,352,574 | 98.80 | 3,182 | 95.61 | 3,305 | | 99.31 |
| >200bp | 3,160 | | 1,326,369 | 98.86 | 3,037 | 96.11 | 3,142 | | 99.43 |
| >500bp | 705 | | 382,437 | 98.85 | 683 | 96.88 | 700 | | 99.29 |
| **Unigene** | | | | | | | | | |
| Dataset | Number | Total  length (bp) | | Covered by assembly (%) | With >90% Sequence in one Scaffold | | | With >50% Sequence in one Scaffold | |
|  |  |  |  |  | Number | Percentage (%) | | Number | Percentage (%) |
| All | 86,222 | 54,249,553 | | 98.97 | 72,882 | 84.53 | | 84,959 | 98.54 |
| >200bp | 86,222 | 54,249,553 | | 98.97 | 72,882 | 84.53 | | 84,959 | 98.54 |
| >500bp | 32,319 | 38,328,599 | | 99.51 | 31,305 | 96.86 | | 32,211 | 99.67 |
| >1 kb | 14,825 | 26,106,917 | | 99.63 | 14,599 | 98.48 | | 14,795 | 99.80 |

**Table S7** Statistical results of the five sequenced fosmid clones aligned to the genome assembly with BLAT

| **Fosmid name** | **Fosmid size(kb)** | **Target**  **name** | **Mismatch (bp)** | **Fosmid gap (bp)** | **Target gap (bp)** | **Match percentage** |
| --- | --- | --- | --- | --- | --- | --- |
| **zzzaxa** | 35.0 | scaffold00036 | 11 | 167 | 41 | 99.5% |
| **zzzbxa** | 33.5 | scaffold00102 | 7 | 406 | 415 | 98.8% |
| **zzzcxa** | 36.8 | scaffold00048 | 2 | 149 | 116 | 99.6% |
| **zzzdxa** | 38.6 | scaffold00024 | 6 | 50 | 54 | 99.9% |
| **zzzexa** | 33.9 | scaffold00008 | 1 | 0 | 72 | 100.0% |
| **Total** | 177.8 | / | 27 | 772 | 698 | 99.6% |

**Table S8** Gene prediction in the sesame genome. Gene sets were predicted independently and then combined to the final gene set, which contained 27,148 protein coding genes.

| **Gene Set** | | **Number** | **Average Transcript Length (bp)** | **Average CDS Length (bp)** | **Average Exon Number**  **per Gene** | **Average Exon Length (bp)** | **Average Intron Length (bp)** |
| --- | --- | --- | --- | --- | --- | --- | --- |
| ***De novo*** | **AUGUSTUS** | 31,127 | 2598.66 | 1161.52 | 5.18 | 224.30 | 343.94 |
|  | **GlimmerHMM** | 36,089 | 2115.66 | 926.43 | 3.82 | 242.67 | 422.07 |
| **Homolog** | ***A. thaliana*** | 22,229 | 2749.17 | 1087.85 | 4.58 | 237.28 | 463.46 |
|  | ***V.vinifera*** | 23,480 | 2987.91 | 1065.69 | 4.85 | 219.53 | 498.71 |
|  | ***R. communis*** | 27,233 | 2407.28 | 977.17 | 4.10 | 238.24 | 461.08 |
|  | ***S. tuberosum*** | 35,365 | 1887.41 | 835.85 | 3.22 | 259.46 | 473.36 |
| **GLEAN** |  | 27,773 | 2821.23 | 1182.11 | 4.76 | 248.46 | 436.20 |
| **RNA_Seq** |  | 27,182 | 3168.96 | 1180.11 | 4.73 | 249.55 | 439.14 |
| **Final Set** |  | 27,148 | 3170.84 | 1180.37 | 4.73 | 249.45 | 439.14 |

Final Set: genes with more than 10% ambiguous bases in CDS region have been filtered.

**Table S9** Number of genes with protein or unigene support

|  | **Number** | **Percentage** |
| --- | --- | --- |
| **Genes with:** |  |  |
| **Protein Support^a^** | 22,585 | 83.19% |
| **Unigene Support^b^** | 16,626 | 61.24% |
| **Protein & Unigene Support** | 15,567 | 57.37% |
| **Protein or Unigene Support** | 23,635 | 87.06% |
| **Ab Initio** | 3,513 | 12.94% |

^a^ Protein database: KEGG, Swiss-Prot, TrEMBL; Protein support criterion: identity ≥ 30%, e value < 1 e-5.

^b^ RNA-Seq clean data was mapped to the genome assembly by TopHat and assembled to unigenes by Cufflinks. For genes show as high as 95% identity and be covered more than 90% by unigenes, we consider they are unigene supported.

**Table S10** Comparison of the gene structure among asterids and rosids clades

|  | **Sesame** | **Potato** | **Tomato** | **Arabidopsis** | **Soybean** | **Poplar** | **Grape** |
| --- | --- | --- | --- | --- | --- | --- | --- |
| Genome assembly size* (Mb) | 273.60 | 682.70 | 737.64 | 119.48 | 955.05 | 403.75 | 470.21 |
| # Genes | 27,148 | 39,031 | 34,763 | 26,637 | 55,787 | 45,033 | 26,346 |
| # Exons | 128,461 | 135,708 | 157,368 | 139,382 | 331,060 | 224,259 | 156,765 |
| # Introns | 101,313 | 96,677 | 122,605 | 112,745 | 275,273 | 179,226 | 130,419 |
| Mean exon per gene | 4.73 | 3.48 | 4.53 | 5.23 | 5.93 | 4.98 | 5.95 |
| Mean exon length (bp) | 249.45 | 266.58 | 228.78 | 237.50 | 206.26 | 231.14 | 191.10 |
| Mean CDS length (bp) | 1180.37 | 926.88 | 1035.65 | 1242.78 | 1224.01 | 1151.06 | 1137.11 |
| Mean intron length (bp) | 439.14 | 621.43 | 540.63 | 157.54 | 423.71 | 347.09 | 969.55 |
| Mean transcripts length (bp) | 3170.84 | 2936.33 | 3163.36 | 1909.57 | 3816.24 | 2916.61 | 6454.02 |

*：Without NNs;

**Table S11** Noncoding genes in the sesame genome

| **Type** | | **Copy Number** | **Average Length (bp)** | **Total Length (bp)** |
| --- | --- | --- | --- | --- |
| **miRNA** | | 207 | 122.73 | 25,405 |
| **tRNA** | | 870 | 75.06 | 65,305 |
| **rRNA** | **rRNA** | 386 | 232.29 | 89,664 |
|  | **18S** | 197 | 344.24 | 67,815 |
|  | **28S** | 124 | 122.91 | 15,241 |
|  | **5.8S** | 33 | 126.88 | 4,187 |
|  | **5S** | 32 | 75.66 | 2,421 |
| **snRNA** | **snRNA** | 268 | 126.60 | 33,930 |
|  | **CD-box** | 118 | 101.88 | 12,022 |
|  | **HACA-box** | 21 | 122.38 | 2,570 |
|  | **splicing** | 129 | 149.91 | 19,338 |

**Table S12** Repeat elements in the sesame genome. Repeat elements were identified by different methods and then combined into the final repeat set. In total, 28.46% of the sesame genome was annotated as repeat elements.

|  | **RepBase TEs** | | **TE Protiens** | | ***De novo*** | | **Combined TEs** | |
| --- | --- | --- | --- | --- | --- | --- | --- | --- |
|  | **Length**  **(bp)** | **%in genome** | **Length (bp)** | **% in genome** | **Length**  **(bp)** | **% in genome** | **Length**  **(bp)** | **% in genome** |
| **DNA** | 2,820,309 | 1.03 | 2,547,265 | 0.93 | 8,079,254 | 2.95 | 10,881,659 | 3.98 |
| **LINE** | 1,192,426 | 0.44 | 7,477,236 | 2.73 | 7,701,075 | 2.82 | 11,571,539 | 4.23 |
| **LTR** | 10,197,999 | 3.73 | 17,262,796 | 6.31 | 39,149933 | 14.31 | 48,030,533 | 17.56 |
| **SINE** | 25,695 | 0.01 | 0 | 0 | 101,023 | 0.04 | 124,172 | 0.05 |
| **Other** | 4,036 | 0 | 0 | 0 | 0 | 0 | 4,036 | 0 |
| **Unknown** | 15,738 | 0.01 | 14,589 | 0.01 | 14,614,303 | 5.34 | 14,643,856 | 5.35 |
| **Total** | 14,006,771 | 5.12 | 27,290,716 | 9.98 | 63,724,637 | 23.29 | 77,856,077 | 28.46 |

**Table S13** Repeat elements in sesame, grape, potato and tomato genomes

|  | **Grape TEs** | | **Potato TEs** | | **Tomato TEs** | | **Sesame TEs** | |
| --- | --- | --- | --- | --- | --- | --- | --- | --- |
| **Type** | **Length (bp)** | **% in genome** | **Length (bp)** | **% in genome** | **Length (bp)** | **% in genome** | **Length**  **(bp)** | **% in genome** |
| **Genome size** | 486,198,630 | / | 727,424,546 | / | 781,666,411 | / | 273,596,034 | / |
| **DNA** | 49,204,348 | 10.12 | 56,153,575 | 7.72 | 36,349,660 | 4.65 | 10,881,659 | 3.98 |
| **LINE** | 23,362,944 | 4.81 | 20,971,834 | 2.88 | 14,097,440 | 1.80 | 11,571,539 | 4.23 |
| **SINE** | 16,287 | 0.00 | 8,248,606 | 1.13 | 3,576,534 | 0.46 | 124,172 | 0.05 |
| **LTR** | 200,658,758 | 41.27 | 358,217,406 | 49.24 | 369,550,553 | 47.28 | 48,030,533 | 17.56 |
| Gypsy | 109,410,515 | 22.50 | 256,807,577 | 35.30 | 274,868,982 | 35.16 | 18,122,609 | 6.62 |
| Copia | 20,059,955 | 4.13 | 74,726,240 | 10.27 | 75,832,093 | 9.70 | 20,059,955 | 7.33 |
| Other | 71,188,288 | 14.64 | 26,683,589 | 3.67 | 18,849,478 | 2.41 | 9,847,969 | 3.60 |
| **Other** | 11,406 | 0.00 | 36,110 | 0.00 | 59,733 | 0.01 | 4,036 | 0.00 |
| **Unknown** | 11,544,277 | 2.37 | 13,470,921 | 1.85 | 25,158,616 | 3.22 | 14,643,856 | 5.35 |
| **Total** | 253,648,279 | 52.17 | 427,417,827 | 58.76 | 421,931,066 | 53.98 | 77,856,077 | 28.46 |

**Table S14** Gene families clustered by OrthoMCL in 11 species

| **Species** | **Total**  **Genes** | **Unclustered**  **Genes** | **Families** | **Unique**  **Families** | **Avg. Genes**  **per Family** |
| --- | --- | --- | --- | --- | --- |
| ***A*. *thaliana*** | 26,637 | 3,664 | 13,298 | 733 | 1.73 |
| ***P*. *trichocarpa*** | 40,303 | 8,013 | 15,108 | 1,090 | 2.14 |
| ***G.. max*** | 42,859 | 4,791 | 14,556 | 1,221 | 2.62 |
| ***O*. *sativa*** | 35,402 | 11,441 | 16,272 | 1,170 | 1.47 |
| ***S*. *bicolor*** | 27,159 | 4,338 | 15,672 | 452 | 1.46 |
| ***M*. *acuminata*** | 34,241 | 8,916 | 12,631 | 688 | 2.00 |
| ***S*. *lycopersicum*** | 33,585 | 7,895 | 17,294 | 505 | 1.49 |
| ***S*. *tuberosum*** | 38,492 | 7,647 | 16,713 | 774 | 1.85 |
| ***V*. *vinifera*** | 25,329 | 6,371 | 13,258 | 646 | 1.43 |
| ***S*. *indicum*** | 27,148 | 3,972 | 13,311 | 450 | 1.74 |
| ***U.gibba*** | 28,025 | 8,564 | 11,695 | 622 | 1.66 |

**Table S15** The duplicated segments of sesame genome corresponding to all 19 grape chromosomes

| Subgenome1 | | | | | | Subgenome2 | | | | | | |
| --- | --- | --- | --- | --- | --- | --- | --- | --- | --- | --- | --- | --- |
| Segments in grape genome | | | Segments in sesame genome | | | Segments in grape genome | | | Segments in sesame genome | | | |
| Chr | Start | End | Chr | Start | End | Chr | Start | End | | Chr | Start | End |
| chr1 | 2,080,886 | 5,272,658 | LG1 | 9,145,814 | 10,531,685 | chr1 | 2,088,886 | 4,015,981 | | LG2 | 17,511,005 | 18,478,987 |
| chr1 | 6,671,069 | 11,265,180 | LG8 | 9,722,181 | 12,158,060 | chr1 | 4,032,048 | 6,605,760 | | LG2 | 14,314,961 | 15,591,613 |
| chr1 | 11,251,118 | 15,307,570 | LG4 | 3,498,495 | 4,586,405 | chr1 | 6,678,843 | 15,323,250 | | LG2 | 15,996,754 | 17,092,617 |
| chr1 | 19,156,487 | 22,797,480 | LG8 | 12,163,253 | 13,659,510 | chr1 | 19,146,130 | 22,211,854 | | LG2 | 15,626,413 | 15,991,389 |
| chr2 | 243,559 | 1,090,707 | LG6 | 6,233,280 | 6,726,512 | chr2 | 213,715 | 1,826,254 | | LG1 | 7,698,359 | 8,578,444 |
| chr2 | 2,810,176 | 5,409,494 | LG6 | 6,740,720 | 9,759,993 | chr2 | 2,804,198 | 4,823,194 | | LG1 | 2,245,471 | 7,658,003 |
| chr2 | 17,148,473 | 18,524,738 | LG6 | 17,247,833 | 17,533,402 | chr2 | 17,306,490 | 18,524,738 | | LG1 | 445,003 | 693,312 |
| chr3 | 78,495 | 2,927,090 | LG10 | 16,397,722 | 17,192,039 | chr3 | 26,344 | 2,962,358 | | LG8 | 20,425,263 | 21,505,398 |
| chr3 | 4,261,888 | 5,903,382 | LG10 | 15,978,067 | 16,322,129 | chr3 | 3,628,918 | 5,903,382 | | LG8 | 19,516,186 | 20,291,293 |
| chr3 | 5,962,731 | 7,389,061 | LG10 | 15,252,890 | 15,546,515 | chr3 | 5,943,312 | 11,346,309 | | LG8 | 18,608,647 | 19,476,739 |
| chr4 | 69,849 | 1,721,410 | LG1 | 1,689,234 | 2,219,262 | chr4 | 69,849 | 2,010,011 | | LG6 | 14,578,940 | 15,495,506 |
| chr4 | 2,736,183 | 4,634,209 | LG1 | 1,259,346 | 1,674,251 | chr4 | 2,657,033 | 4,612,704 | | LG6 | 15,502,598 | 16,359,593 |
| chr4 | 6,537,828 | 9,364,925 | LG1 | 852,681 | 1,171,578 | chr4 | 4,689,101 | 5,739,296 | | LG6 | 14,223,489 | 14,576,129 |
| chr4 | 16,253,492 | 17,370,254 | LG4 | 16,184,031 | 16,502,151 | chr4 | 6,448,272 | 9,364,925 | | LG6 | 16,446,606 | 16,905,706 |
| chr4 | 18,547,351 | 19,343,256 | LG6 | 22,928,699 | 23,387,601 | chr4 | 16,120,243 | 17,385,152 | | LG7 | 9,692,687 | 9,887,946 |
|  |  |  |  |  |  | chr4 | 17,675,368 | 18,546,815 | | LG15 | 5,385,649 | 5,822,854 |
|  |  |  |  |  |  | chr4 | 19,652,828 | 20,711,649 | | LG15 | 7,034,966 | 7,702,609 |
|  |  |  |  |  |  | chr4 | 21,277,236 | 23,356,942 | | LG15 | 6,326,813 | 7,019,263 |
| chr5 | 1,307,369 | 1,911,416 | LG10 | 12,735 | 214,027 | chr5 | 262,346 | 1,793,585 | | LG3 | 211,232 | 723,017 |
| chr5 | 2,906,822 | 14,544,632 | LG10 | 215,060 | 4,203,549 | chr5 | 2,972,568 | 5,383,593 | | LG3 | 737,753 | 1,990,732 |
| chr5 | 24,266,597 | 24,901,872 | LG7 | 10,245,395 | 10,416,784 | chr5 | 5,436,715 | 9,176,001 | | LG3 | 14,434,965 | 15,843,560 |
|  |  |  |  |  |  | chr5 | 9,179,172 | 17,468,305 | | LG3 | 16,693,842 | 17,817,667 |
|  |  |  |  |  |  | chr5 | 23,226,800 | 24,843,489 | | LG3 | 18,297,553 | 19,375,559 |
| chr6 | 318,230 | 911,945 | LG9 | 4,302,402 | 4,472,629 | chr6 | 149,444 | 1,223,731 | | LG9 | 1,528,651 | 1,776,628 |
| chr6 | 1,905,407 | 2,651,983 | LG9 | 7,741,952 | 7,937,890 | chr6 | 1,249,275 | 2,888,750 | | LG6 | 3,201,060 | 4,016,771 |
| chr6 | 3,012,711 | 6,375,974 | LG9 | 1,986,158 | 3,413,047 | chr6 | 3,142,691 | 6,873,091 | | LG9 | 4,666,168 | 5,665,919 |
| chr6 | 10,159,255 | 17,564,065 | LG9 | 6,624,778 | 7,302,211 | chr6 | 7,937,326 | 9,402,123 | | LG5 | 13,350,259 | 14,557,734 |
| chr6 | 17,590,085 | 19,533,564 | LG9 | 5,677,803 | 6,466,117 | chr6 | 15,076,740 | 17,564,065 | | LG6 | 4,028,063 | 4,785,465 |
| chr6 | 19,537,178 | 21,362,550 | LG9 | 7,432,511 | 7,737,712 | chr6 | 17,935,258 | 21,505,147 | | LG9 | 47,381 | 971,010 |
| chr7 | 59,086 | 688,584 | LG6 | 2,020,203 | 2,274,492 | chr7 | 323,573 | 5,167,581 | | LG6 | 18,706 | 2,139,732 |
| chr7 | 422,306 | 4,302,931 | LG6 | 18,422,813 | 19,914,350 | chr7 | 5,849,605 | 11,464,831 | | LG6 | 2,323,227 | 3,169,887 |
| chr7 | 5,701,995 | 11,464,831 | LG6 | 22,137,235 | 22,785,958 | chr7 | 15,310,139 | 16,207,035 | | LG15 | 3,798,760 | 4,177,098 |
| chr7 | 15,589,461 | 16,681,877 | LG13 | 2,814,457 | 3,069,783 | chr7 | 16,242,881 | 17,053,374 | | LG15 | 2,595,847 | 3,080,256 |
| chr8 | 7,395,825 | 10,919,437 | LG11 | 14,692,645 | 15,326,447 | chr8 | 7,688,742 | 10,444,884 | | LG5 | 980,325 | 1,176,649 |
| chr8 | 12,481,948 | 16,310,409 | LG11 | 13,080,808 | 14,686,225 | chr8 | 11,203,024 | 12,398,746 | | LG5 | 18,181,407 | 18,928,651 |
| chr8 | 13,520,106 | 14,247,891 | LG11 | 13,515,540 | 13,851,988 | chr8 | 12,481,948 | 14,690,550 | | LG5 | 248,490 | 875,063 |
| chr8 | 16,361,299 | 18,342,392 | LG11 | 11,039,748 | 12,217,485 | chr8 | 16,439,784 | 17,728,345 | | LG5 | 1,665,327 | 2,192,539 |
| chr8 | 18,353,680 | 18,991,320 | LG11 | 12,820,037 | 13,065,382 | chr8 | 18,416,283 | 18,996,818 | | LG5 | 56,966 | 245,855 |
| chr8 | 19,963,759 | 21,067,868 | LG6 | 4,837,114 | 5,230,526 | chr8 | 19,963,759 | 21,034,111 | | LG4 | 58,982 | 606,388 |
| chr8 | 21,152,385 | 22,372,476 | LG6 | 5,238,732 | 5,622,249 | chr8 | 21,172,463 | 21,941,417 | | LG4 | 614,676 | 1,037,677 |
| chr9 | 56,559 | 6,552,732 | LG3 | 7,011,566 | 9,687,930 | chr9 | 146,979 | 10,538,433 | | LG1 | 11,139,387 | 12,524,882 |
| chr9 | 6,657,638 | 10,608,381 | LG3 | 13,921,369 | 14,429,703 |  |  |  | |  |  |  |
| chr10 | 132,655 | 1,256,368 | LG8 | 8,581,453 | 8,987,445 | chr10 | 507,800 | 1,176,476 | | LG12 | 3,176,866 | 3,364,177 |
| chr10 | 1,336,331 | 2,949,126 | LG8 | 7,737,501 | 8,013,200 | chr10 | 1,288,720 | 2,565,368 | | LG12 | 4,212,861 | 4,787,313 |
| chr10 | 3,000,367 | 11,909,157 | LG8 | 152,051 | 795,447 | chr10 | 3,915,070 | 11,642,515 | | LG12 | 4,802,798 | 5,799,941 |
| chr11 | 5,145,835 | 8,395,698 | LG7 | 5,107,879 | 6,840,014 | chr11 | 5,951,957 | 7,468,337 | | LG5 | 8,165,398 | 9,591,635 |
| chr11 | 13,642,812 | 17,749,621 | LG11 | 9,826,572 | 10,542,252 | chr11 | 7,893,567 | 13,795,228 | | LG5 | 19,548,065 | 20,505,307 |
| chr11 | 17,897,335 | 19,781,001 | LG2 | 9,497,621 | 10,451,805 | chr11 | 13,995,151 | 17,728,584 | | LG5 | 2,518,161 | 3,032,415 |
|  |  |  |  |  |  | chr11 | 17,936,593 | 19,699,333 | | LG5 | 19,013,514 | 19,519,142 |
| chr12 | 16,762,995 | 22,592,055 | LG8 | 16,755,546 | 17,768,185 | chr12 | 14,705,726 | 22,662,359 | | LG10 | 13,775,984 | 14,825,729 |
| chr13 | 154,715 | 1,789,660 | LG4 | 11,819,905 | 13,061,085 | chr13 | 154,715 | 1,808,939 | | LG7 | 10,902,518 | 11,571,338 |
| chr13 | 3,314,624 | 4,557,770 | LG4 | 13,082,560 | 13,666,616 | chr13 | 3,135,518 | 4,557,770 | | LG7 | 10,515,340 | 10,891,498 |
| chr13 | 20,037,047 | 24,390,809 | LG1 | 15,030,476 | 16,221,770 | chr13 | 18,585,556 | 22,074,913 | | LG10 | 6,093,886 | 7,918,404 |
| chr14 | 29,846 | 2,870,678 | LG8 | 15,615,646 | 16,711,701 | chr14 | 116,202 | 2,482,009 | | LG10 | 12,027,270 | 13,700,824 |
| chr14 | 16,421,913 | 22,023,785 | LG15 | 4,701,581 | 5,376,343 | chr14 | 17,430,423 | 22,046,642 | | LG13 | 1,993,889 | 2,635,837 |
| chr14 | 22,568,777 | 24,295,609 | LG15 | 2,089,023 | 2,571,192 | chr14 | 22,124,290 | 24,215,135 | | LG8 | 2,850,932 | 3,391,422 |
| chr14 | 24,592,270 | 26,516,806 | LG15 | 8,503 | 711,304 | chr14 | 24,569,426 | 27,610,473 | | LG13 | 3,078,514 | 3,672,242 |
| chr14 | 26,916,660 | 30,252,880 | LG15 | 763,069 | 2,056,462 | chr14 | 27,646,939 | 29,948,299 | | LG8 | 3,289,149 | 3,971,320 |
| chr15 | 9,799,164 | 11,460,454 | LG1 | 14,214,642 | 14,517,329 | chr15 | 8,617,002 | 11,256,521 | | LG11 | 6,118,101 | 6,867,280 |
| chr15 | 11,522,706 | 16,169,795 | LG1 | 16,244,936 | 17,496,658 | chr15 | 11,211,122 | 14,583,448 | | LG11 | 2,233,392 | 4,906,200 |
| chr15 | 15,163,744 | 15,959,136 | LG1 | 17,166,613 | 17,385,914 | chr15 | 16,574,044 | 20,253,423 | | LG11 | 51,126 | 1,349,506 |
| chr15 | 16,926,728 | 20,268,488 | LG1 | 17,511,843 | 18,529,312 |  |  |  | |  |  |  |
| chr16 | 5,068,577 | 20,816,151 | LG4 | 9,137,892 | 11,676,562 | chr16 | 16,208,718 | 21,237,766 | | LG7 | 8,532,839 | 9,352,056 |
| chr16 | 21,004,457 | 21,867,415 | LG4 | 8,109,159 | 8,692,735 | chr16 | 21,010,799 | 21,978,336 | | LG7 | 9,089,765 | 9,457,872 |
| chr17 | 47,749 | 2,767,609 | LG1 | 12,927,462 | 14,163,917 | chr17 | 109,248 | 2,540,667 | | LG3 | 11,517,457 | 13,162,788 |
| chr17 | 5,802,295 | 8,199,083 | LG8 | 14,554,840 | 15,477,315 | chr17 | 5,827,095 | 6,124,614 | | LG3 | 2,816,862 | 3,420,151 |
| chr17 | 8,466,343 | 9,201,391 | LG8 | 14,208,491 | 14,529,538 | chr17 | 6,151,295 | 7,055,235 | | LG3 | 2,014,441 | 2,872,572 |
|  |  |  |  |  |  | chr17 | 7,060,940 | 8,381,026 | | LG3 | 9,985,861 | 10,809,489 |
|  |  |  |  |  |  | chr17 | 8,239,693 | 13,863,458 | | LG3 | 3,311,064 | 6,074,807 |
| chr18 | 99,836 | 944,601 | LG3 | 19,422,589 | 19,784,681 | chr18 | 331,414 | 992,640 | | LG2 | 3,071,200 | 3,737,842 |
| chr18 | 1,204,422 | 1,782,971 | LG3 | 23,755,686 | 23,878,256 | chr18 | 978,609 | 1,444,174 | | LG2 | 3,763,960 | 4,038,022 |
| chr18 | 1,811,442 | 3,832,303 | LG3 | 24,176,873 | 24,594,113 | chr18 | 1,792,259 | 3,369,305 | | LG2 | 4,740,955 | 5,447,881 |
| chr18 | 4,048,925 | 13,554,243 | LG3 | 20,097,385 | 24,101,930 | chr18 | 3,351,205 | 3,832,303 | | LG2 | 1,065,184 | 1,353,937 |
| chr18 | 12,985,919 | 16,217,640 | LG3 | 23,915,203 | 24,160,251 | chr18 | 3,562,717 | 5,218,160 | | LG2 | 1,216,864 | 2,588,716 |
|  |  |  |  |  |  | chr18 | 6,893,423 | 8,324,703 | | LG2 | 7,312,564 | 9,054,264 |
|  |  |  |  |  |  | chr18 | 8,266,901 | 12,971,832 | | LG7 | 2,516,869 | 5,295,819 |
|  |  |  |  |  |  | chr18 | 11,726,652 | 12,691,517 | | LG7 | 2,651,850 | 3,122,294 |
|  |  |  |  |  |  | chr18 | 12,964,305 | 16,233,137 | | LG2 | 4,291,744 | 4,727,922 |
| chr19 | 48,560 | 3,858,658 | LG14 | 3,766,816 | 4,865,333 | chr19 | 48,560 | 3,267,133 | | LG6 | 19,952,626 | 20,775,565 |
| chr19 | 4,111,005 | 10,749,212 | LG14 | 160,791 | 1,729,012 | chr19 | 3,286,207 | 10,749,212 | | LG12 | 1,697 | 1,908,164 |
| chr19 | 22,323,288 | 23,888,873 | LG8 | 9,270,289 | 9,450,229 | chr19 | 18,712,666 | 23,873,669 | | LG12 | 1,931,531 | 2,468,380 |

**Table S16** Gene retention in the two subgenomes of sesame. The two subgenomes were derived from recent whole genome duplication (WGD) event.

| Gene loss and retention after recent WGD in sesame | | Number of sesame ancestral gene loci | Number of sesame genes retained after recent WGD |
| --- | --- | --- | --- |
| 1:1 (grapevine: sesame) | retained in Subgenome 1 | 2,422 (40.8%)* | 2,422 (33.7%) |
|  | retained in Subgenome 2 | 2,280 (38.3%) | 2,280 (31.8%) |
| Total |  | 2,702 (79.1%) |  |
| 1:2 (grapevine: sesame) | Two copies both retained | 1,239 (20.9%) | 2,478 (34.5%) |
| Total | | 5,941 (100%) | 7,180 (100%) |

*Percentage of the loci to total.

This table was summed up from Data S5 in additional file 2.

**Table S17** The gene fractionation depth in the sesame genome

| Genomic loci for Grapevine: Sesame | (a) | (b) | (c) | (d) | (e) | (f) |
| --- | --- | --- | --- | --- | --- | --- |
| 1:1 | 6423 (75.96%) | 6391  (75.25%) | 6235  (75.77%) | 6125  (75.9%) | 5965  (76.6%) | 5856  (76.9%) |
| 1:2 | 1948 | 1959 | 1847 | 1788 | 1686 | 1614 |
| 1:3 | 82 | 126 | 127 | 134 | 119 | 121 |
| 1:4 | 2 | 15 | 18 | 19 | 17 | 16 |
| 1:5 | 0 | 2 | 2 | 4 | 3 | 2 |

We used MCscan (<http://chibba.agtec.uga.edu/duplication/mcscan>) with a series of gradually loose parameters (a)-(f) to construct grape-sesame syntenic blocks in consideration of the high degree of fractionation of gamma (γ)-derived segments due to long evolutionary time and repeated fractionation affected by the following recent WGD in sesame.

(a) MATCH_SIZE: **5**; UNIT_DIST: **2**; OVERLAP_WINDOW: **8**; # EXTENSION_DIST: **40.**

(b) MATCH_SIZE: **5**; UNIT_DIST: **4**; OVERLAP_WINDOW: **16**; # EXTENSION_DIST: **80**

(c) MATCH_SIZE: **5**; UNIT_DIST: **8**; OVERLAP_WINDOW: **32**; # EXTENSION_DIST: **160**

(d) MATCH_SIZE: **5**; UNIT_DIST: **10**; OVERLAP_WINDOW: **40**; # EXTENSION_DIST: **200**

(e) MATCH_SIZE: **5**; UNIT_DIST: **15**; OVERLAP_WINDOW: **60**; # EXTENSION_DIST: **300**

(f) MATCH_SIZE: **5**; UNIT_DIST: **20**; OVERLAP_WINDOW: **80**; # EXTENSION_DIST: **400**

**Table S18** Significantly enriched GO terms of duplicated genes from recent whole genome duplication (WGD) in the sesame genome

|  | **GO_ID** | **GO_Term** | **GO_Class** | **AdjustedPv** | **2- copies retained genes** | **Whole genome** |
| --- | --- | --- | --- | --- | --- | --- |
| **Transport** | GO:0006810 | Transport | BP | 1.572E-04 | 212 | 1384 |
|  | GO:0006811 | ion transport | BP | 3.299E-04 | 68 | 357 |
|  | GO:0015031 | protein transport | BP | 4.198E-02 | 48 | 281 |
|  | GO:0006812 | cation transport | BP | 1.329E-02 | 54 | 304 |
|  | GO:0046907 | intracellular transport | BP | 7.221E-03 | 45 | 234 |
|  | GO:0030001 | metal ion transport | BP | 5.985E-03 | 35 | 168 |
|  | GO:0015672 | monovalent inorganic cation transport | BP | 4.580E-02 | 26 | 132 |
|  | GO:0015992 | proton transport | BP | 3.785E-02 | 16 | 68 |
|  | GO:0006820 | anion transport | BP | 5.591E-03 | 14 | 43 |
|  | GO:0015991 | ATP hydrolysis coupled proton transport | BP | 9.898E-04 | 14 | 37 |
|  | GO:0033177 | proton-transporting two-sector ATPase complex, proton-transporting domain | CC | 1.282E-03 | 10 | 21 |
|  | GO:0015746 | citrate transport | BP | 2.080E-02 | 3 | 3 |
|  | GO:0015137 | citrate transmembrane transporter activity | MF | 2.080E-02 | 3 | 3 |
|  | GO:0033179 | proton-transporting V-type ATPase, V0 domain | CC | 2.637E-02 | 4 | 6 |
| **Regulation** |  |  |  |  |  |  |
|  | GO:0065007 | biological regulation | BP | 6.254E-09 | 261 | 1565 |
|  | GO:0050789 | regulation of biological process | BP | 6.254E-09 | 257 | 1534 |
|  | GO:0050794 | regulation of cellular process | BP | 4.943E-09 | 248 | 1455 |
|  | GO:0019222 | regulation of metabolic process | BP | 3.721E-07 | 202 | 1210 |
|  | GO:0060255 | regulation of macromolecule metabolic process | BP | 3.663E-08 | 190 | 1094 |
|  | GO:0019219 | regulation of nucleobase, nucleoside, nucleotide and nucleic acid metabolic process | BP | 1.919E-08 | 193 | 1107 |
|  | GO:0010468 | regulation of gene expression | BP | 1.919E-08 | 189 | 1074 |
|  | GO:0045449 | regulation of transcription | BP | 1.565E-08 | 188 | 1059 |
|  | GO:0003700 | sequence-specific DNA binding transcription factor activity | MF | 2.080E-02 | 85 | 534 |
|  | GO:0010467 | gene expression | BP | 3.057E-02 | 240 | 1753 |
|  | GO:0006350 | transcription | BP | 4.964E-07 | 195 | 1164 |
|  | GO:0000156 | two-component response regulator activity | MF | 2.637E-02 | 12 | 42 |
|  | GO:0019887 | protein kinase regulator activity | MF | 1.190E-02 | 6 | 11 |
|  | GO:0016538 | cyclin-dependent protein kinase regulator activity | MF | 4.435E-02 | 4 | 7 |
| **Transduction** |  |  |  |  |  |  |
|  | GO:0007165 | signal transduction | BP | 1.046E-02 | 49 | 266 |
|  | GO:0000160 | two-component signal transduction system (phosphorelay) | BP | 2.080E-02 | 14 | 53 |
|  | GO:0009725 | response to hormone stimulus | BP | 1.046E-02 | 10 | 27 |
|  | GO:0004428 | inositol or phosphatidylinositol kinase activity | MF | 1.046E-02 | 10 | 27 |
|  | GO:0016307 | phosphatidylinositol phosphate kinase activity | MF | 4.662E-03 | 8 | 16 |
| **Metabolic** |  |  |  |  |  |  |
|  | GO:0043170 | macromolecule metabolic process | BP | 1.925E-02 | 514 | 3978 |
|  | GO:0044238 | primary metabolic process | BP | 9.530E-03 | 665 | 5205 |
|  | GO:0006139 | nucleobase, nucleoside, nucleotide and nucleic acid metabolic process | BP | 3.362E-02 | 253 | 1861 |
|  | GO:0090304 | nucleic acid metabolic process | BP | 4.175E-02 | 220 | 1606 |
|  | GO:0072527 | pyrimidine-containing compound metabolic process | BP | 3.911E-02 | 7 | 19 |
|  | GO:0019637 | organophosphate metabolic process | BP | 3.702E-02 | 15 | 62 |
|  | GO:0017111 | nucleoside-triphosphatase activity | MF | 1.192E-02 | 108 | 691 |
|  | GO:0016462 | pyrophosphatase activity | MF | 1.188E-02 | 110 | 705 |
|  | GO:0016818 | hydrolase activity, acting on acid anhydrides, in phosphorus-containing anhydrides | MF | 9.580E-03 | 113 | 721 |

Note: Chi-square test or Fisher test (when n<5) were conducted in the matrix data: the 2 copies retained genes in each GO term (column 5), all genes in each GO term (column 6), the 2 copies retained genes with GO annotation (1,658), all genes with GO annotation (14,396). FDR method was used to adjust the final P-value. BP: biological process; CC: cellular component; MF: molecular function.

**Table S19** Disease resistance proteins in sesame, potato, tomato and grape genomes

| **Type** | **Sesame** | **Potato** | **Tomato** | **Grape** |
| --- | --- | --- | --- | --- |
| **TIR-NBS** | 0 | 15 | 8 | 3 |
| **TIR-NBS-LRR** | 0 | 29 | 16 | 17 |
| **CC-NBS** | 25 | 44 | 18 | 18 |
| **CC-NBS-LRR** | 5 | 7 | 4 | 28 |
| **NBS-LRR** | 23 | 55 | 21 | 121 |
| **NBS** | 118 | 286 | 188 | 129 |
| **Total** | 171 | 436 | 255 | 316 |

**Table S**20 Diversity levels of sesame and other species' populations

|  | Cultivated | | | | |
| --- | --- | --- | --- | --- | --- |
|  | Sesame | Watermelon | Soybean | Chickpea | Rice |
| *π* (10^-3^) | 2.5075 | 1.4188 | 1.894 | 2.000 | 5.400 |
| *θw* (10^-3^) | 3.0012 | 1.5254 | 1.689 | 1.798 | 6.600 |

**Supplementary Figures**


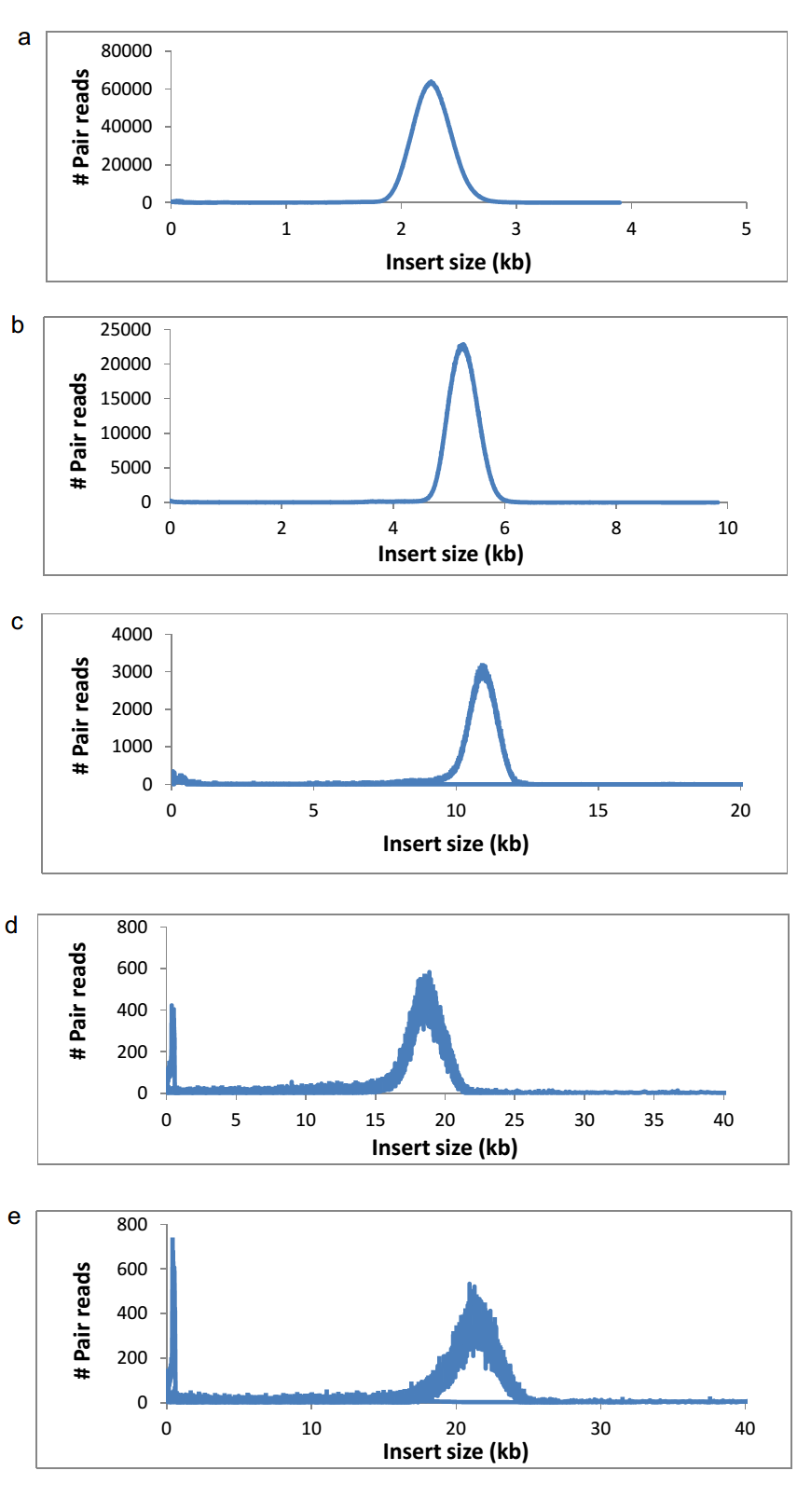


**Figure S1** Distributions of the clean reads generated from the long-insert libraries. (a) 2 kb insert library; (b) 5 kb insert library; (c) 10 kb insert library; (d) The first 20 kb insert library; (e) The second 20 kb insert library. The distributions of these reads showed the six long-insert libraries were constructed successfully.

**Figure S2** *k*-mer analysis to estimate the sesame genome size. The figure shows frequency of 17 *k*-mers which are 17 bp sequences from the reads (after filtering) of short-insert size libraries. We identified 12,482,678,912 *k*-mers using 15.75 Gb data. The genome size can be estimated by (total *k*-mer number) / (the volume peak), which was thus estimated as 357 Mb.


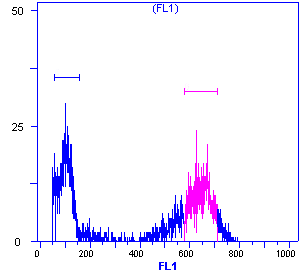


**Reference** erythrocytes mon erythrocytes

**Sesame** erythrocytes mon erythrocytes

| Region | FL1 Mean | Pct Gated | Pct Total | FL1 HPCV | Pct Total |
| --- | --- | --- | --- | --- | --- |
| Sesame | 102.9 | 36.88% | 12.27% | 3.11% | 12.27% |
| Reference | 645.7 | 37.18% | 12.37% | 0.93% | 12.37% |

**Figure S3** Flow cytometric analysis of the genome size of sesame. Salmon erythrocytes (2.16pg/1C) was used as internal biological reference. The C-value of sesame was estimated to be 0.34pg/1C.


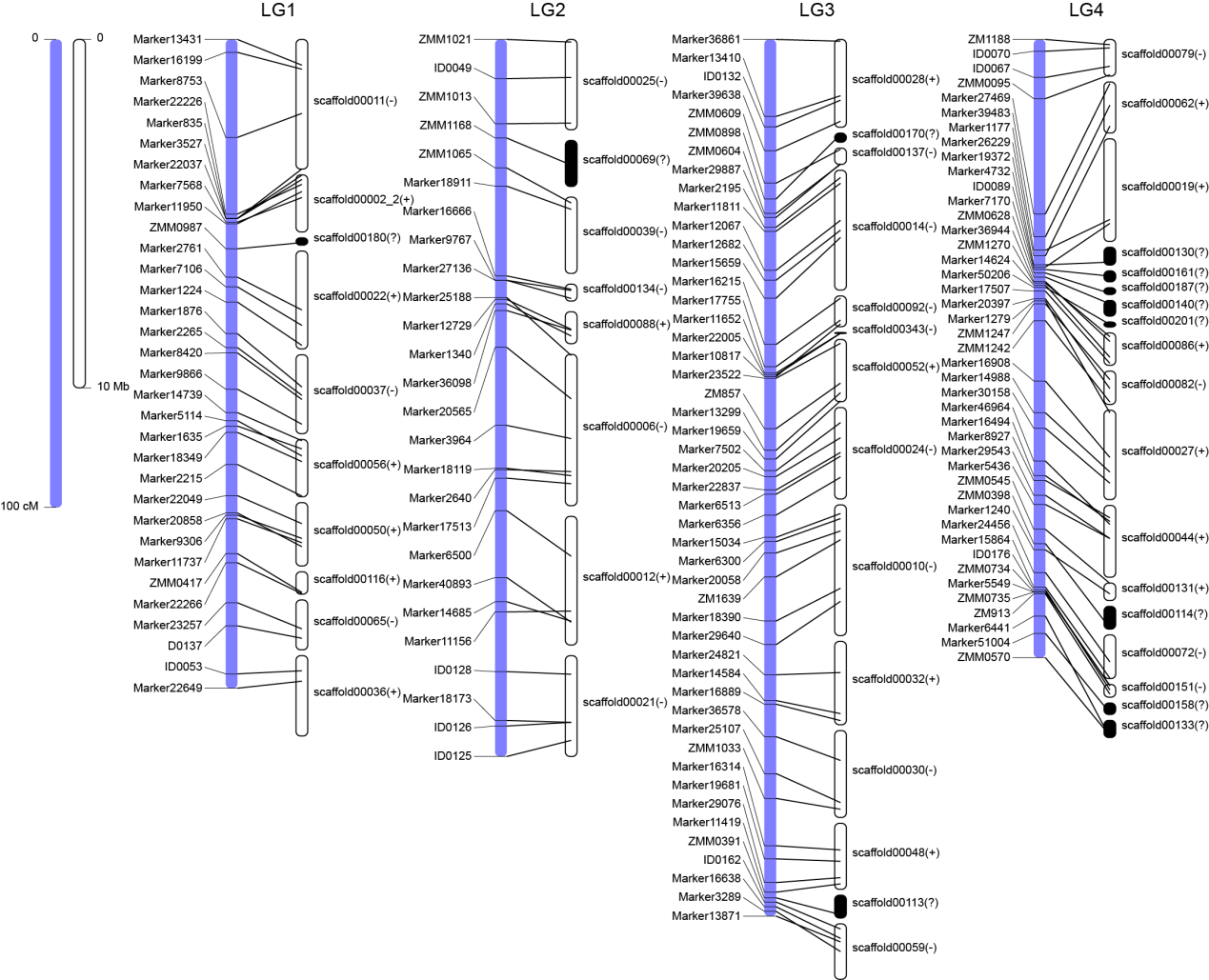


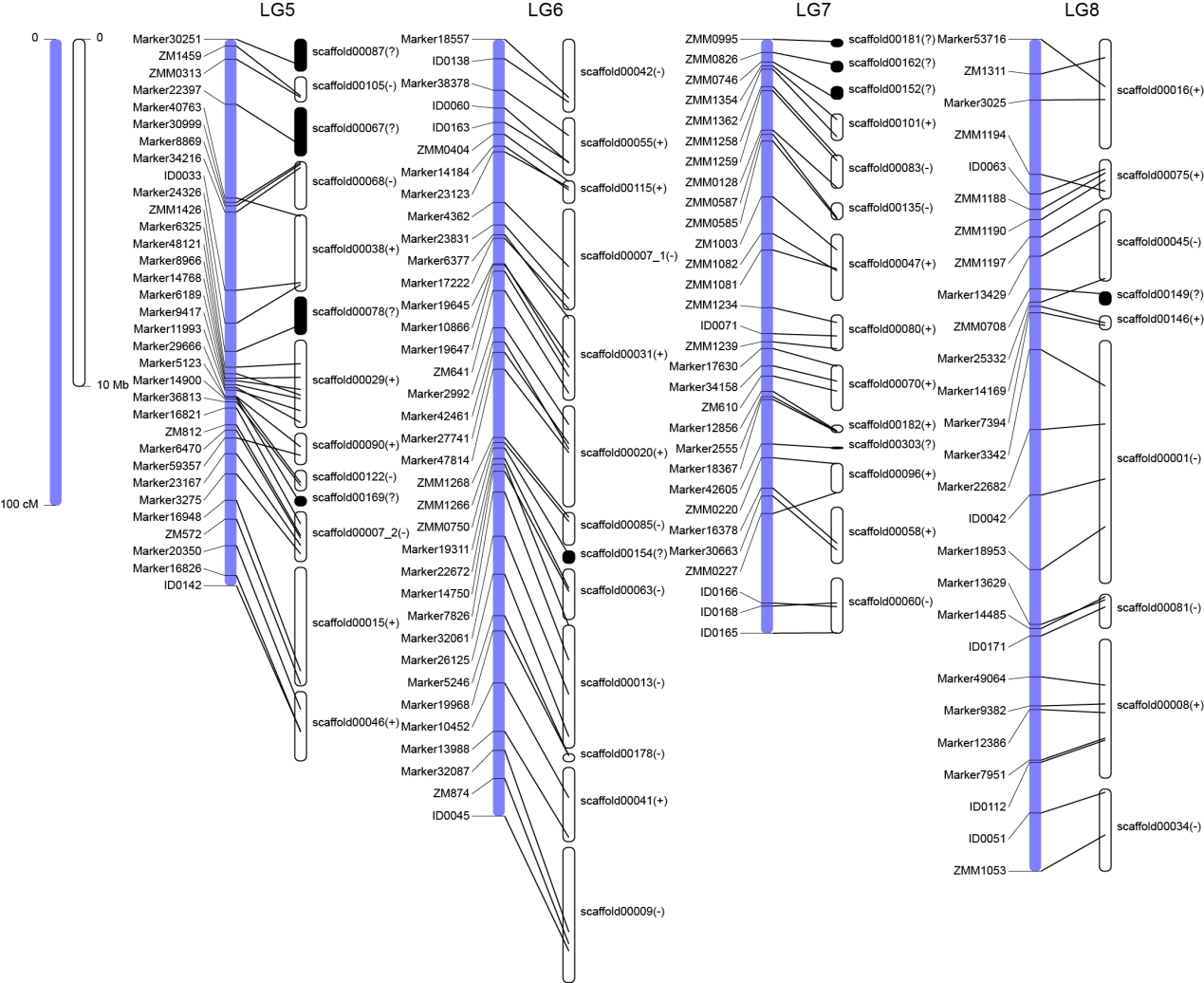


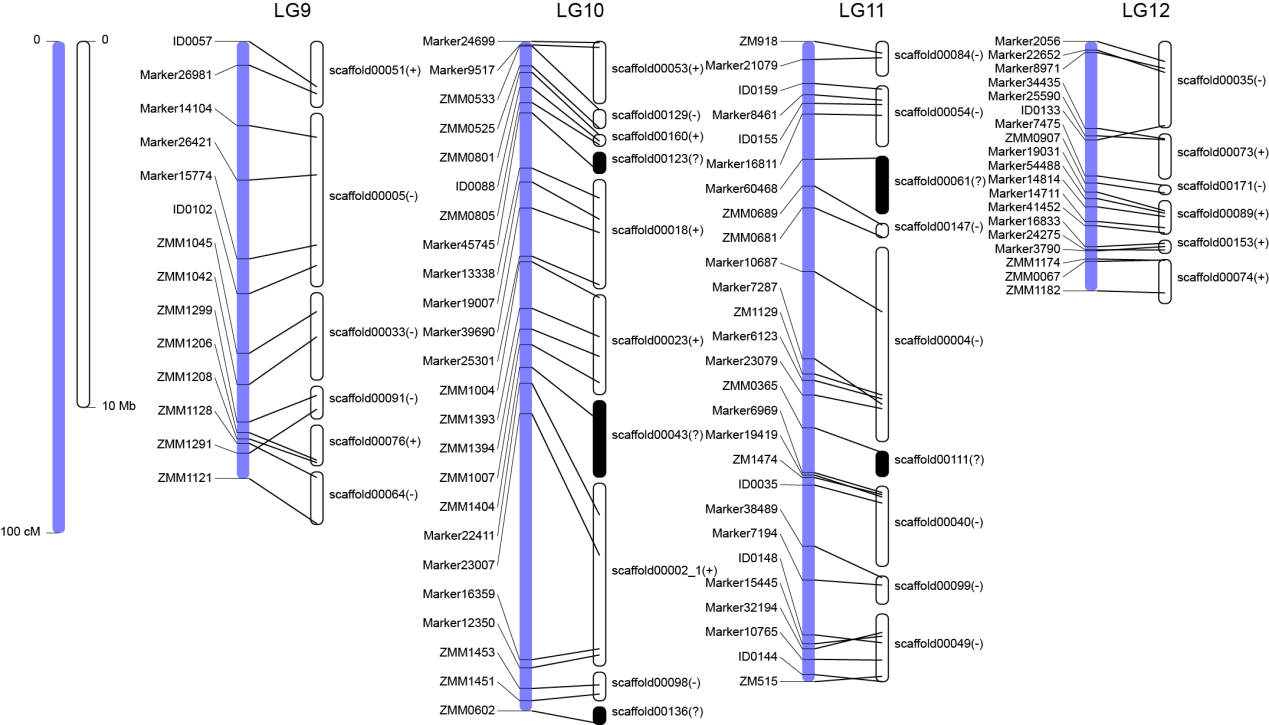

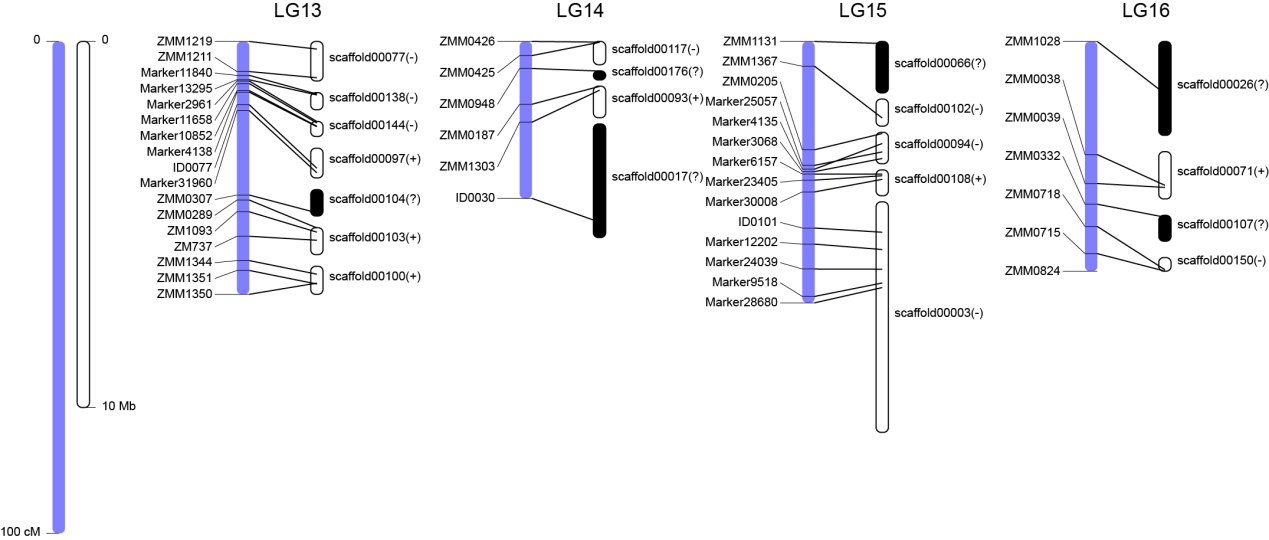


**Figure S4** Map of the sequence scaffolds along the sesame linkage groups (LGs). The linkage groups are represented as blue bars on the left. The sequence scaffolds are represented on the right as white bars (orientated) or black bars (random orientation).


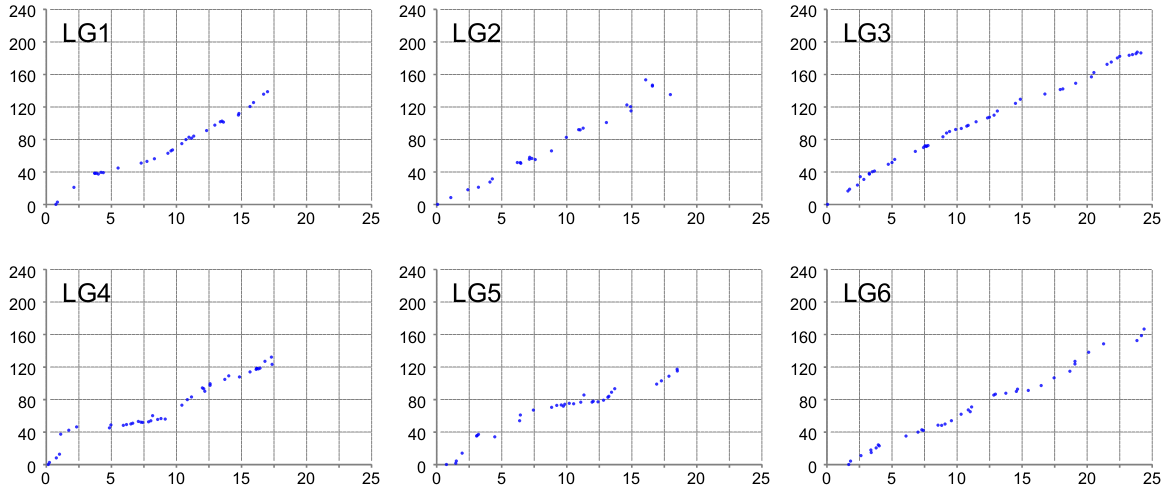

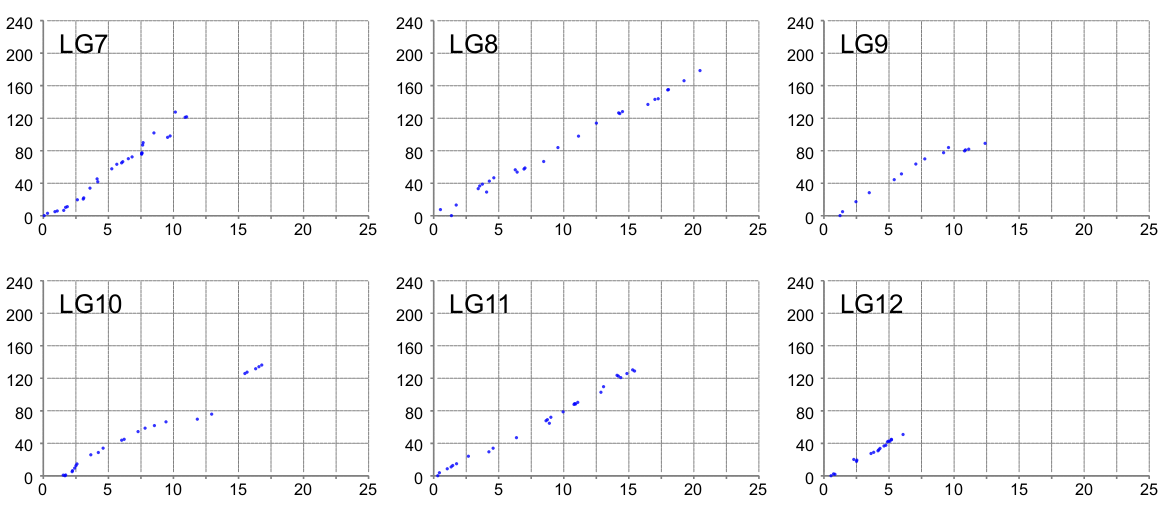

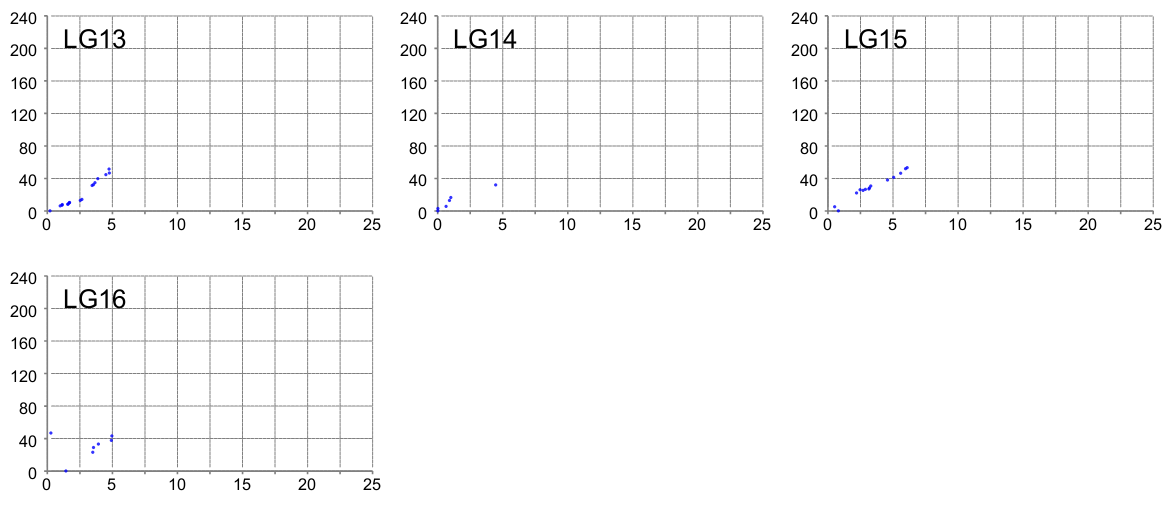


cM

Mb

**Figure S5** Genetic distance vs physical distance. Genetic position of the 403 genetic markers was plotted against the corresponding physical position.

**Figure S6** The GC content distributions of sesame and other sequenced plants

**Figure S7** Nucleotide alignments of five sequenced fosmids from sesame to their corresponding scaffold regions in the Illumina assembly. The top red tracks represent fosmids, and the bottom blue tracks show scaffolds. The orange shading between the scaffold and fosmid tracks represents areas of at least 90% nucleotide identity. White regions on the scaffold tracks indicate NNs regions in the assembled sequences.

**Figure S8** Distribution of the insertion time of long terminal repeats (LTRs) in sesame

**Figure S9** Distribution of the divergence rates of LTRs. The divergence rate was calculated between the identified TE elements in the genome and the consensus sequence in the TE library built by *de novo* methods.

**
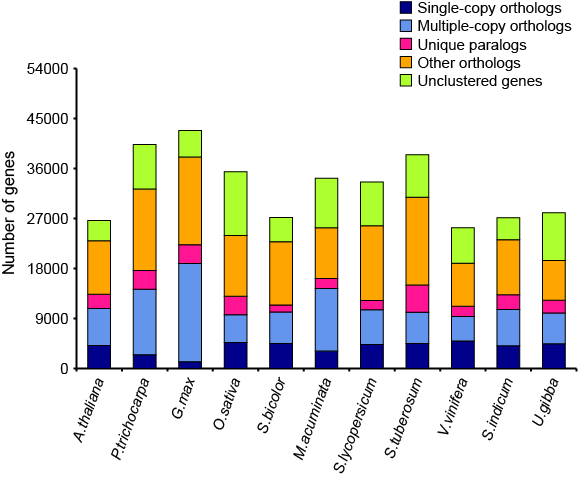
**

**Figure S10** Gene number in each category defined by OrthoMCL


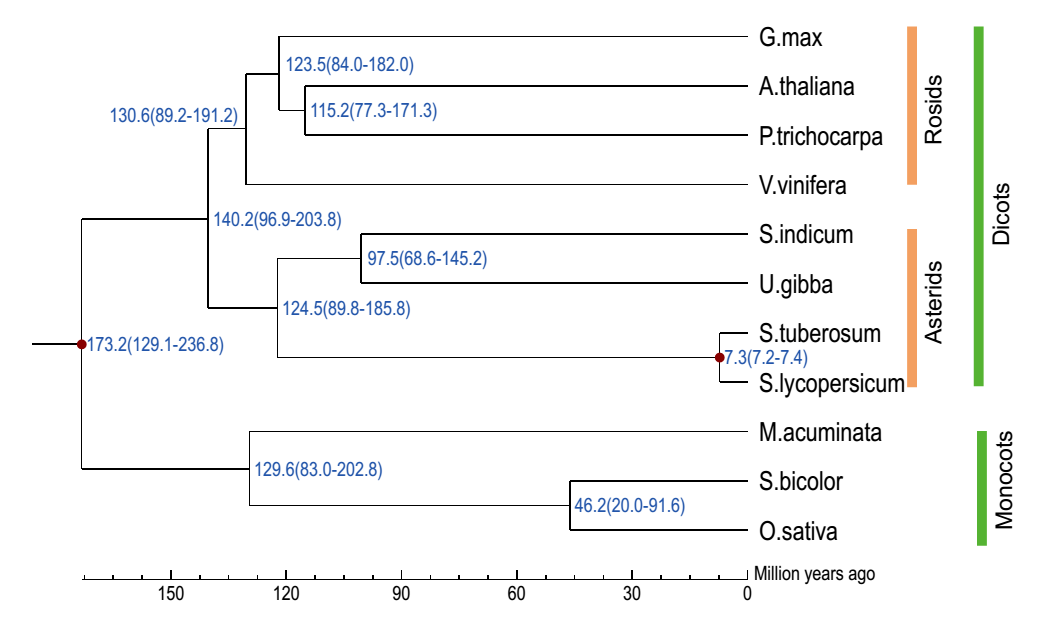


**Figure S11** The phylogenetic relationship and split-time estimation based on all single-copy gene families shared by all species used


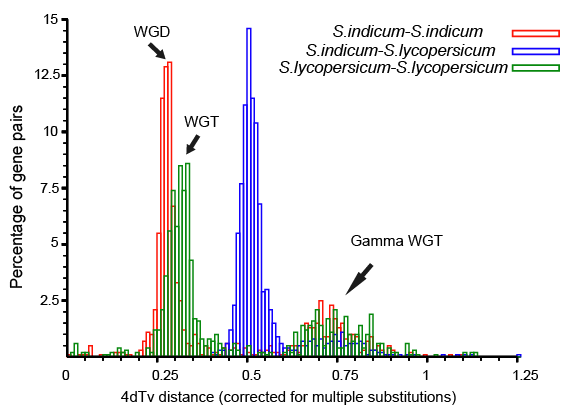


**Figure S12** Distribution of the 4dTv distance between duplicated genes of syntenic regions in sesame (red bar) and tomato (green bar). The blue bar shows the 4dTv divergence of orthologous gene pairs between sesame and tomato.

**Figure S13** The *K*_s_ (synonymous) (x-axis) and *K*_a_/*K*_s_ (y-axis) distribution for each syntenic block in the sesame genome. Each dot represents the average *K*_s_ and *K*_a_/*K*_s_ value of all duplicated genes in a block.


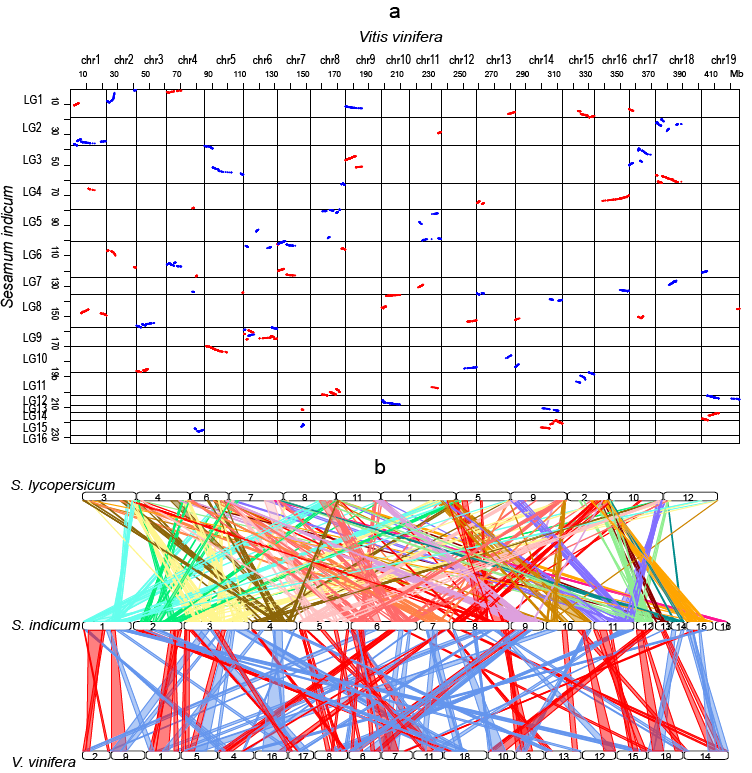


**Figure S14** Two subgenomes originated from the ancestral WGD of the sesame genome were identified using the grape genome as reference. (a) The dot plot for comparing the sesame and grape genomes. (b) Syntenic blocks between grapevine (V. vinifera), tomato (S. lycopersicum), and sesame (S. indicum). Syntenic blocks between sesame and tomato were constructed based on reciprocal best hits of gene pairs. The two subgenome regions from sesame corresponding to grapevine are colored red and blue, respectively.

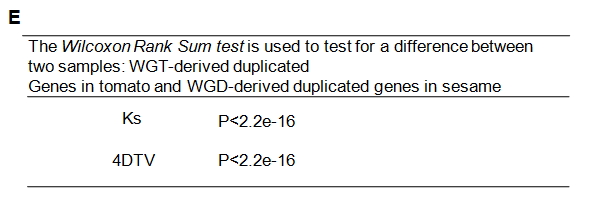


**Figure S15** Distributions of the Ks (A and B) and 4DTV (C and D) of the duplicated genes in sesame and tomato. These genes were derived from the WGT event in tomato and recent WGD in sesame, respectively. The Wilcoxon Rank Sum test is used to test for a difference between two samples (E).


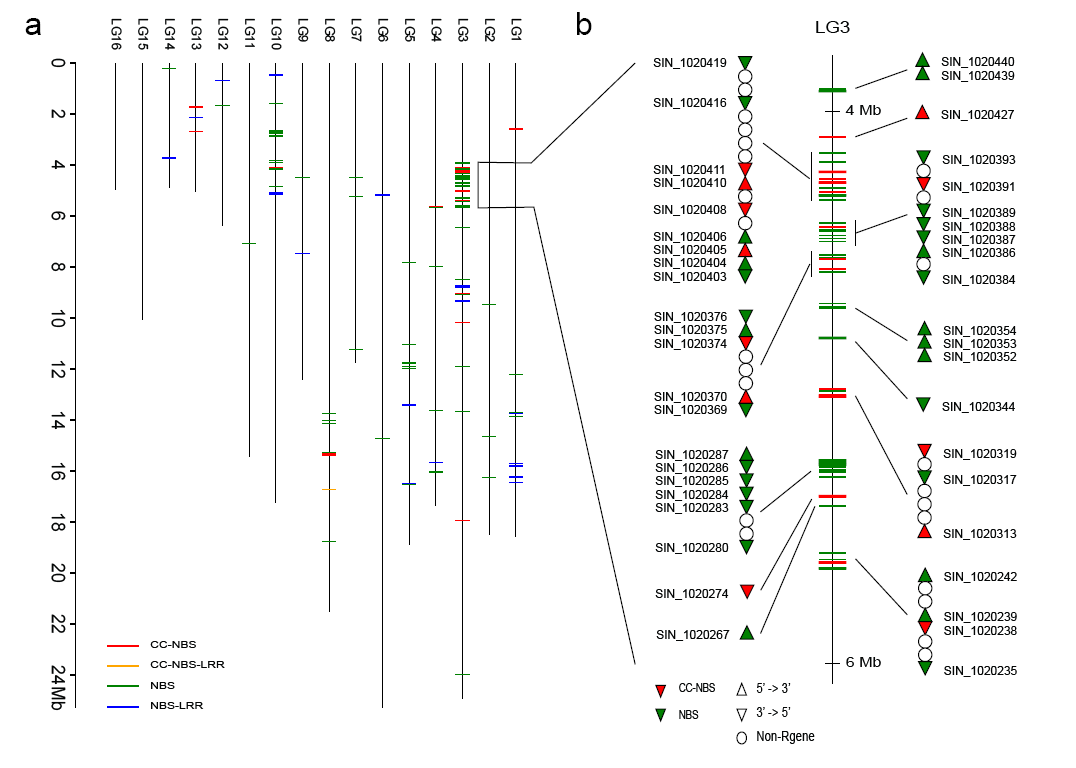


**Figure S16** Distributions of nucleotide-binding site (NBS)-encoding resistance gene models along sesame linkage groups. (a) Distribution of the 171 R-genes of different types along 16 sesame linkage groups. These genes are denoted with short color lines, and many of them are arranged in tandem arrays. (b) Detailed overview of R-gene clusters on LG3 from 3.9 to 5.8 Mb in sesame.

**Figure S17** Phylogenetic analysis of TIR-type NBS-encoding gene homologues belonging to the same OrthoMCL group generated from 10 species. Monophyletic clades are collapsed into filled triangles, with numbers at the base of the triangle indicating the number of genes in the given clade. Sesame and monocots (rice, sorghum, banana) were absent from this group, in contrast to a clear expansion in poplar and soybean. Gray, poplar; green, soybean; purple, grape; black, *Arabidopsis thaliana*; olive, potato; red, tomato.


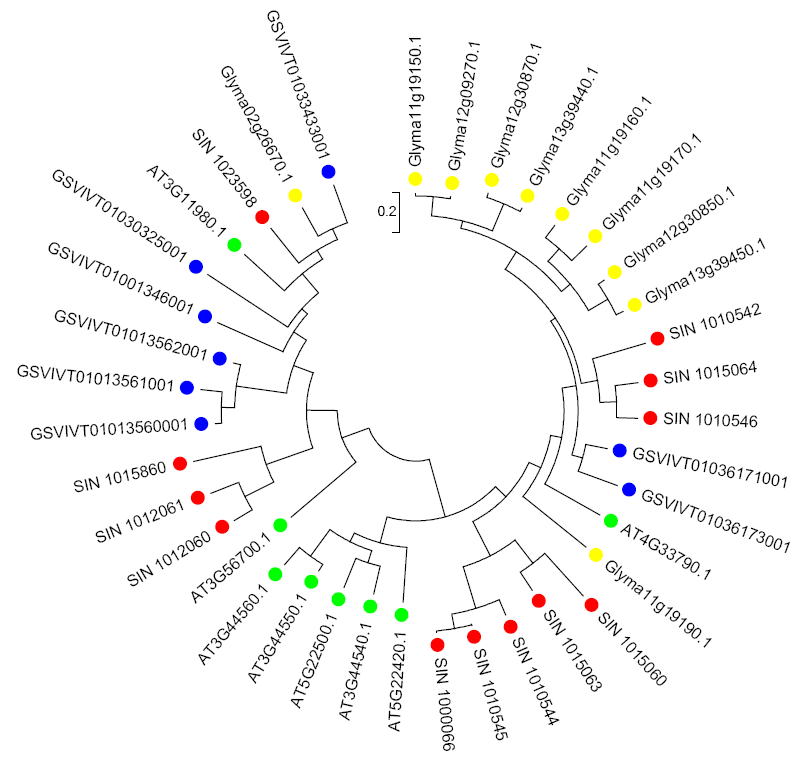


**Figure S18** Phylogenetic tree of the alcohol-forming fatty acyl-CoA reductase (AlcFAR) gene family. Sesame (red), soybean (yellow), *A. thaliana* (green) and grape (blue) genes were shown in the tree with corresponding genome ID nomenclature respectively.


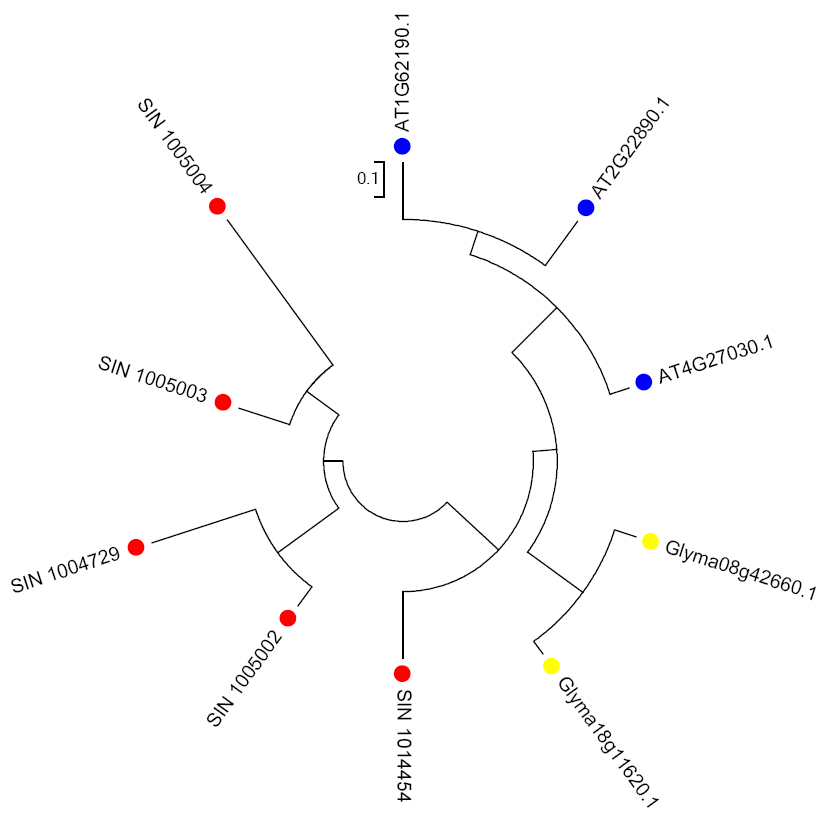


**Figure S19** Phylogenetic tree of the FAD4-like desaturase (FAD4 like) gene family. Sesame (red), soybean (yellow), *A. thaliana* (green) and grape (blue) genes were shown in the tree with corresponding genome ID nomenclature respectively.


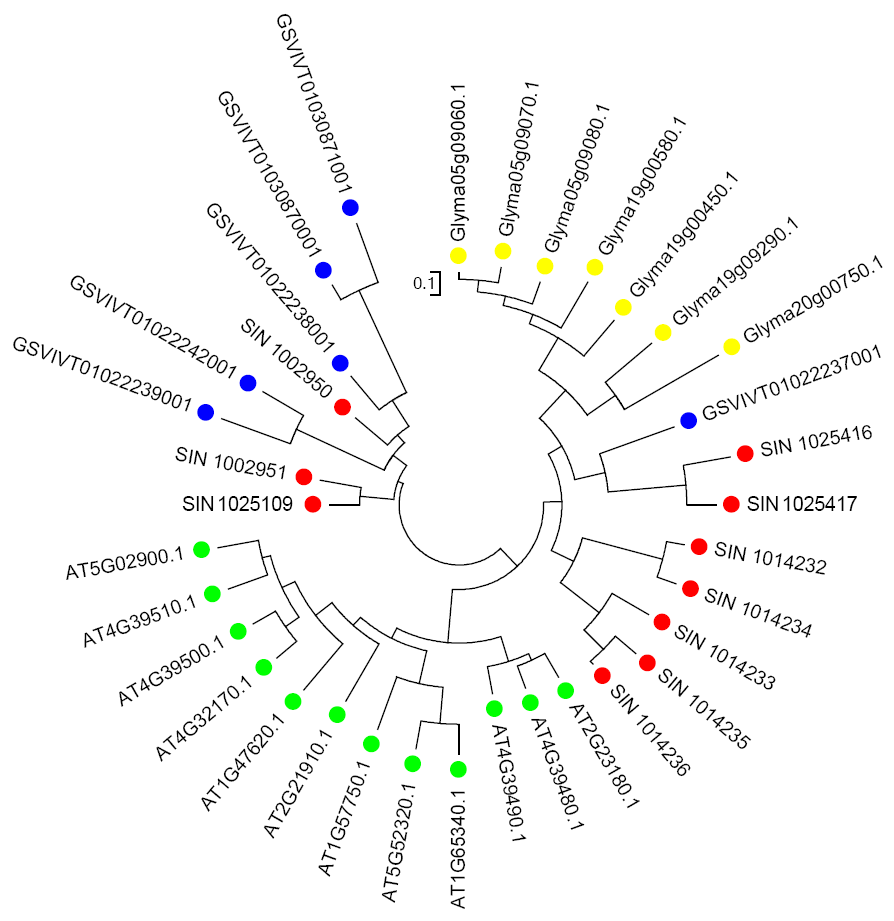


**Figure S20** Phylogenetic tree of the midchain alkane hydroxylase gene family. Sesame (red), soybean (yellow), *A. thaliana* (green) and grape (blue) genes were shown in the tree with corresponding genome ID nomenclature respectively.


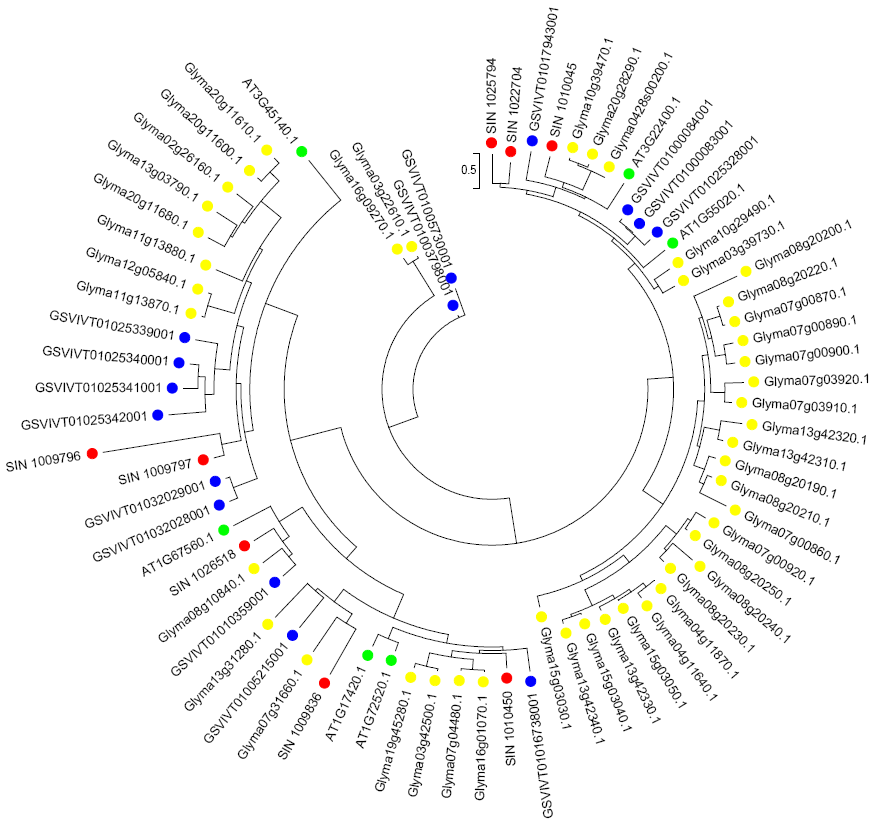


**Figure S21** Phylogenetic tree of the lipoxygenase (LOX) gene family. Sesame (red), soybean (yellow), *A. thaliana* (green) and grape (blue) genes were shown in the tree with corresponding genome ID nomenclature respectively.


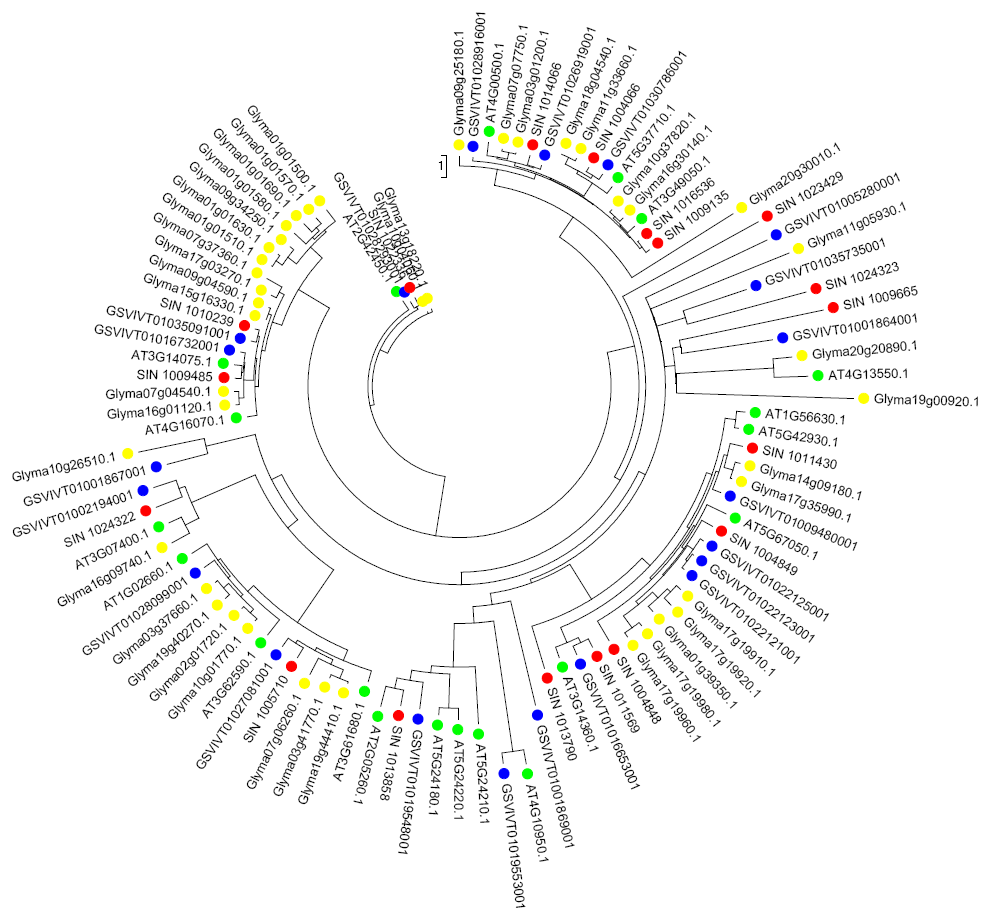


**Figure S22** Phylogenetic tree of the lipid acyl hydrolase-like (LAH) gene family. Sesame (red), soybean (yellow), *A. thaliana* (green) and grape (blue) genes were shown in the tree with corresponding genome ID nomenclature respectively.


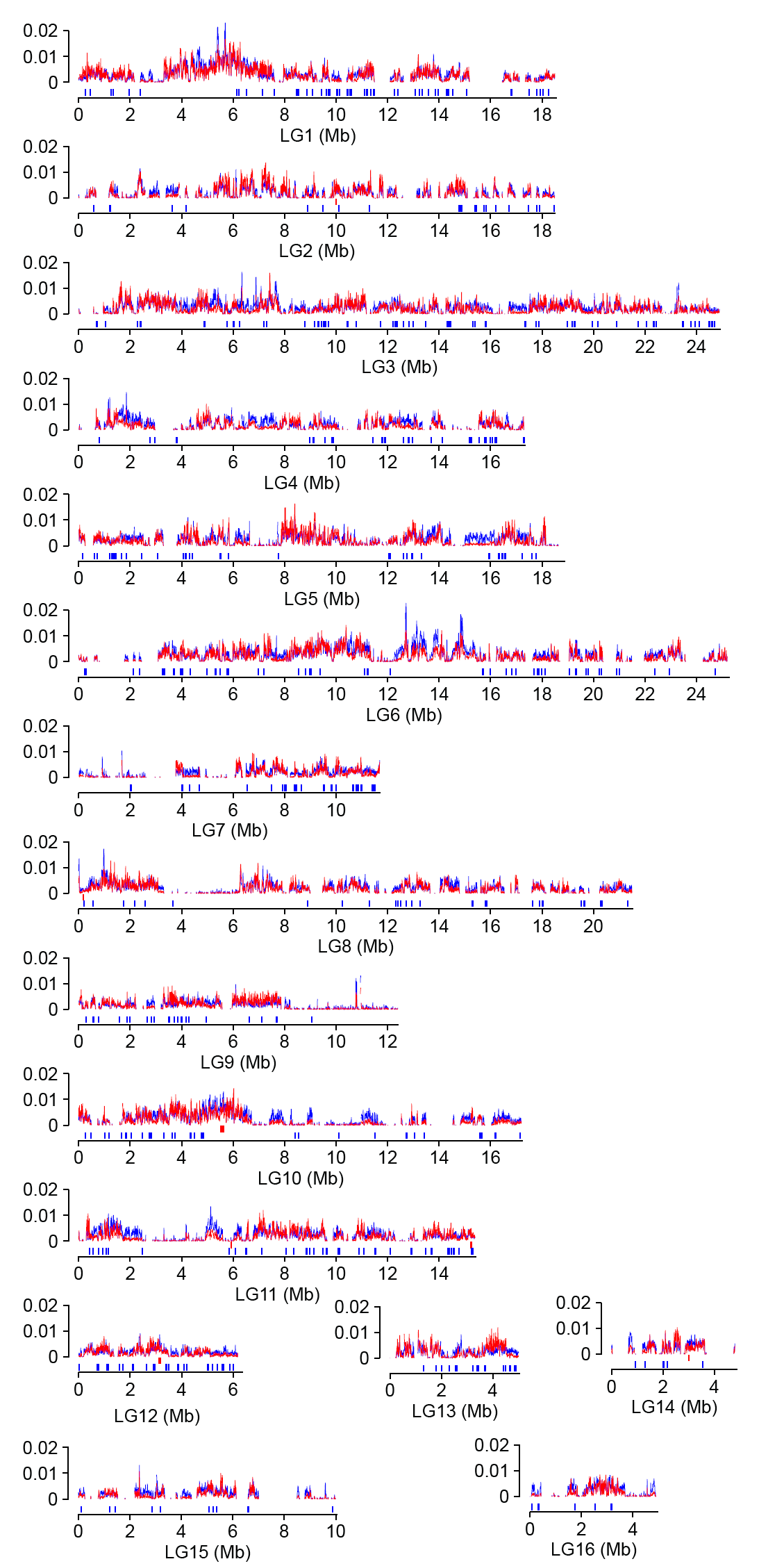


**Figure S23** Distributions of π (red) and θw (blue) of the sesame genome and the positions of lipid- related genes. The two lines of bars below the axis of π or θw show the positions of the lipid related genes in sesame. Blue bars, lipid related genes except for LTP1; Red bars, LTP1 genes.


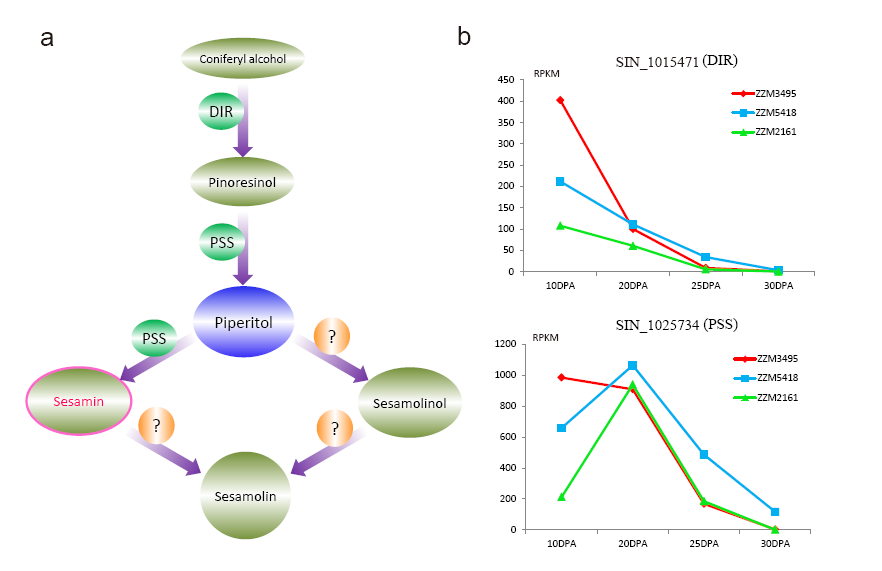


**Figure S24** Expression patterns of the key genes involved in the sesamin biosynthesis pathway. (**a**) The pathway of sesamin biosynthesis from coniferyl alcohol. The green ovals indicate the key genes DIR and PSS. (**b**) The expression patterns of the DIR (upper panel, SIN_1015471) and PSS (lower panel, SIN_1025734) genes in the three sesame accessions ZZM3495 (sesamin content: 1.1% of seed), ZZM5418 (sesamin content: 0.4% of seed) and ZZM2161 (sesamin content: 0.1% of seed).


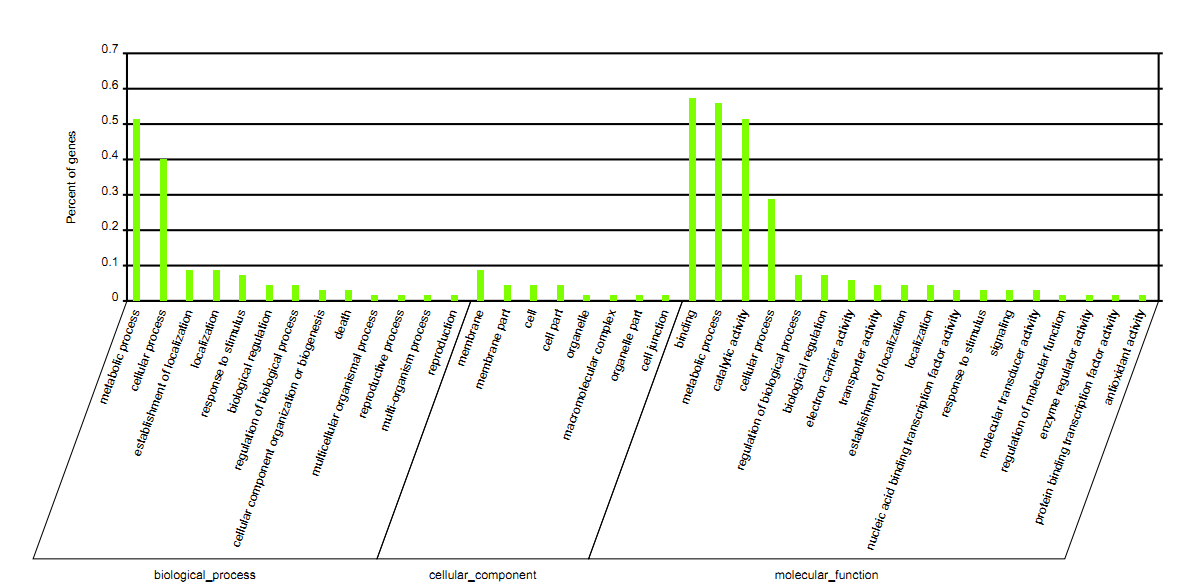


**Figure** **S25** GO distribution of the genes correlated with (Pearson's correlation coefficients > 0.9) PSS (SIN_1025734).

References

1. Doyle JJ, Doyle JL: **Isolation of plant DNA from fresh tissue.** *Focus* 1990**:**13-15.

2. Li R, Zhu H, Ruan J, Qian W, Fang X, Shi Z, Li Y, Li S, Shan G, Kristiansen K, et al: **De novo assembly of human genomes with massively parallel short read sequencing.** *Genome Res* 2010, **20:**265-272.

3. Wang X, Wang H, Wang J, Sun R, Wu J, Liu S, Bai Y, Mun JH, Bancroft I, Cheng F, et al: **The genome of the mesopolyploid crop species Brassica rapa.** *Nat Genet* 2011, **43:**1035-1039.

4. Simpson JT, Wong K, Jackman SD, Schein JE, Jones SJ, Birol I: **ABySS: a parallel assembler for short read sequence data.** *Genome Res* 2009, **19:**1117-1123.

5. Li R, Fan W, Tian G, Zhu H, He L, Cai J, Huang Q, Cai Q, Li B, Bai Y, et al: **The sequence and de novo assembly of the giant panda genome.** *Nature* 2010, **463:**311-317.

6. Huang S, Li R, Zhang Z, Li L, Gu X, Fan W, Lucas WJ, Wang X, Xie B, Ni P, et al: **The genome of the cucumber, *Cucumis sativus* L.** *Nat Genet* 2009, **41:**1275-1281.

7. Zhang G, Liu X, Quan Z, Cheng S, Xu X, Pan S, Xie M, Zeng P, Yue Z, Wang W, et al: **Genome sequence of foxtail millet (*Setaria italica*) provides insights into grass evolution and biofuel potential.** *Nat Biotechnol* 2012, **30:**549-554.

8. Dolezel J, Greilhuber J, Suda J: **Estimation of nuclear DNA content in plants using flow cytometry.** *Nat Protocols* 2007, **2:**2233-2244.

9. Galbraith DW, Harkins KR, Maddox JM, Ayres NM, Sharma DP, Firoozabady E: **Rapid flow cytometric analysis of the cell cycle in intact plant tissues.** *Science* 1983, **220:**1049-1051.

10. Pfosser M, Amon A, Lelley T, Heberle-Bors E: **Evaluation of sensitivity of flow cytometry in detecting aneuploidy in wheat using disomic and ditelosomic wheat-rye addition lines.** *Cytometry* 1995, **21:**387-393.

11. Dolezel J, Bartos J, Voglmayr H, Greilhuber J: **Nuclear DNA content and genome size of trout and human.** *Cytometry Part A* 2003, **51:**127-128; author reply 129.

12. Jirimutu, Wang Z, Ding G, Chen G, Sun Y, Sun Z, Zhang H, Wang L, Hasi S, Zhang Y, et al: **Genome sequences of wild and domestic bactrian camels.** *Nat Commun* 2012, **3:**1202.

13. Varshney RK, Chen W, Li Y, Bharti AK, Saxena RK, Schlueter JA, Donoghue MT, Azam S, Fan G, Whaley AM, et al: **Draft genome sequence of pigeonpea (*Cajanus cajan*), an orphan legume crop of resource-poor farmers.** *Nat Biotechnol* 2012, **30:**83-89.

14. Li H, Durbin R: **Fast and accurate short read alignment with Burrows-Wheeler transform.** *Bioinformatics* 2009, **25:**1754-1760.

15. Li H, Handsaker B, Wysoker A, Fennell T, Ruan J, Homer N, Marth G, Abecasis G, Durbin R: **The Sequence Alignment/Map format and SAMtools.** *Bioinformatics* 2009, **25:**2078-2079.

16. Schuler GD: **Sequence mapping by electronic PCR.** *Genome Res* 1997, **7:**541-550.

17. Suh MC, Kim MJ, Hur CG, Bae JM, Park YI, Chung CH, Kang CW, Ohlrogge JB: **Comparative analysis of expressed sequence tags from *Sesamum indicum* and *Arabidopsis thaliana* developing seeds.** *Plant Mol Biol* 2003, **52:**1107-1123.

18. Kent WJ: **BLAT--the BLAST-like alignment tool.** *Genome Res* 2002, **12:**656-664.

19. Wei W, Qi X, Wang L, Zhang Y, Hua W, Li D, Lv H, Zhang X: **Characterization of the sesame (*Sesamum indicum* L.) global transcriptome using Illumina paired-end sequencing and development of EST-SSR markers.** *BMC Genomics* 2011, **12:**451.

20. Arabidopsis Genome Initiative: **Analysis of the genome sequence of the flowering plant Arabidopsis thaliana.** *Nature* 2000, **408:**796-815.

21. Jaillon O, Aury JM, Noel B, Policriti A, Clepet C, Casagrande A, Choisne N, Aubourg S, Vitulo N, Jubin C, et al: **The grapevine genome sequence suggests ancestral hexaploidization in major angiosperm phyla.** *Nature* 2007, **449:**463-467.

22. Chan AP, Crabtree J, Zhao Q, Lorenzi H, Orvis J, Puiu D, Melake-Berhan A, Jones KM, Redman J, Chen G, et al: **Draft genome sequence of the oilseed species *Ricinus communis*.** *Nat Biotechnol* 2010, **28:**951-956.

23. Xu X, Pan S, Cheng S, Zhang B, Mu D, Ni P, Zhang G, Yang S, Li R, Wang J, et al: **Genome sequence and analysis of the tuber crop potato.** *Nature* 2011, **475:**189-195.

24. Birney E, Durbin R: **Using GeneWise in the Drosophila annotation experiment.** *Genome Res* 2000, **10:**547-548.

25. Stanke M, Keller O, Gunduz I, Hayes A, Waack S, Morgenstern B: **AUGUSTUS: ab initio prediction of alternative transcripts.** *Nucleic Acids Res* 2006, **34:**W435-439.

26. Majoros WH, Pertea M, Salzberg SL: **TigrScan and GlimmerHMM: two open source ab initio eukaryotic gene-finders.** *Bioinformatics* 2004, **20:**2878-2879.

27. Trapnell C, Pachter L, Salzberg SL: **TopHat: discovering splice junctions with RNA-Seq.** *Bioinformatics* 2009, **25:**1105-1111.

28. Hunter S, Apweiler R, Attwood TK, Bairoch A, Bateman A, Binns D, Bork P, Das U, Daugherty L, Duquenne L, et al: **InterPro: the integrative protein signature database.** *Nucleic Acids Res* 2009, **37:**D211-215.

29. Mistry J, Finn R: **Pfam: a domain-centric method for analyzing proteins and proteomes.** *Methods Mol Biol* 2007, **396:**43-58.

30. Attwood TK, Beck ME, Bleasby AJ, Parry-Smith DJ: **PRINTS--a database of protein motif fingerprints.** *Nucleic Acids Res* 1994, **22:**3590-3596.

31. Hulo N, Bairoch A, Bulliard V, Cerutti L, De Castro E, Langendijk-Genevaux PS, Pagni M, Sigrist CJ: **The PROSITE database.** *Nucleic Acids Res* 2006, **34:**D227-230.

32. Bru C, Courcelle E, Carrere S, Beausse Y, Dalmar S, Kahn D: **The ProDom database of protein domain families: more emphasis on 3D.** *Nucleic Acids Res* 2005, **33:**D212-215.

33. Schultz J, Milpetz F, Bork P, Ponting CP: **SMART, a simple modular architecture research tool: identification of signaling domains.** *Proc Natl Acad Sci U S A* 1998, **95:**5857-5864.

34. Ashburner M, Ball CA, Blake JA, Botstein D, Butler H, Cherry JM, Davis AP, Dolinski K, Dwight SS, Eppig JT, et al: **Gene ontology: tool for the unification of biology. The Gene Ontology Consortium.** *Nat Genet* 2000, **25:**25-29.

35. Kanehisa M, Goto S: **KEGG: kyoto encyclopedia of genes and genomes.** *Nucleic Acids Res* 2000, **28:**27-30.

36. Lowe TM, Eddy SR: **tRNAscan-SE: a program for improved detection of transfer RNA genes in genomic sequence.** *Nucleic Acids Res* 1997, **25:**955-964.

37. Xu Z, Wang H: **LTR_FINDER: an efficient tool for the prediction of full-length LTR retrotransposons.** *Nucleic Acids Res* 2007, **35:**W265-268.

38. Edgar RC, Myers EW: **PILER: identification and classification of genomic repeats.** *Bioinformatics* 2005, **21:**i152-158.

39. Price AL, Jones NC, Pevzner PA: **De novo identification of repeat families in large genomes.** *Bioinformatics* 2005, **21 Suppl 1:**i351-358.

40. Tarailo-Graovac M, Chen N: **Using RepeatMasker to identify repetitive elements in genomic sequences.** *Curr Protoc Bioinformatics* 2009, **4**.

41. Jurka J, Kapitonov VV, Pavlicek A, Klonowski P, Kohany O, Walichiewicz J: **Repbase Update, a database of eukaryotic repetitive elements.** *Cytogenet Genome Res* 2005, **110:**462-467.

42. Benson G: **Tandem repeats finder: a program to analyze DNA sequences.** *Nucleic Acids Res* 1999, **27:**573-580.

43. McCarthy EM, McDonald JF: **LTR_STRUC: a novel search and identification program for LTR retrotransposons.** *Bioinformatics* 2003, **19:**362-367.

44. Edgar RC: **MUSCLE: multiple sequence alignment with high accuracy and high throughput.** *Nucleic Acids Res* 2004, **32:**1792-1797.

45. Labbe J, Murat C, Morin E, Tuskan GA, Le Tacon F, Martin F: **Characterization of transposable elements in the ectomycorrhizal fungus Laccaria bicolor.** *PLoS One* 2012, **7:**e40197.

46. Li L, Stoeckert CJ, Jr., Roos DS: **OrthoMCL: identification of ortholog groups for eukaryotic genomes.** *Genome Res* 2003, **13:**2178-2189.

47. Guindon S, Dufayard JF, Lefort V, Anisimova M, Hordijk W, Gascuel O: **New algorithms and methods to estimate maximum-likelihood phylogenies: assessing the performance of PhyML 3.0.** *Syst Biol* 2010, **59:**307-321.

48. Hasegawa M, Kishino H, Yano T: **Dating of the human-ape splitting by a molecular clock of mitochondrial DNA.** *Journal of Molecular Evolution* 1985, **22:**160-174.

49. Shulaev V, Sargent DJ, Crowhurst RN, Mockler TC, Folkerts O, Delcher AL, Jaiswal P, Mockaitis K, Liston A, Mane SP, et al: **The genome of woodland strawberry (*Fragaria vesca*).** *Nat Genet* 2011, **43:**109-116.

50. Anisimova M, Gascuel O: **Approximate likelihood-ratio test for branches: A fast, accurate, and powerful alternative.** *Syst Biol* 2006, **55:**539-552.

51. Wehe A, Bansal MS, Burleigh JG, Eulenstein O: **DupTree: a program for large-scale phylogenetic analyses using gene tree parsimony.** *Bioinformatics* 2008, **24:**1540-1541.

52. Yang Z: **PAML 4: phylogenetic analysis by maximum likelihood.** *Mol Biol Evol* 2007, **24:**1586-1591.

53. The Tomato Genome Consortium: **The tomato genome sequence provides insights into fleshy fruit evolution.** *Nature* 2012, **485:**635-641.

54. McDonnell AV, Jiang T, Keating AE, Berger B: **Paircoil2: improved prediction of coiled coils from sequence.** *Bioinformatics* 2006, **22:**356-358.

55. Moreno-Hagelsieb G, Latimer K: **Choosing BLAST options for better detection of orthologs as reciprocal best hits.** *Bioinformatics* 2008, **24:**319-324.

56. Koonin EV: **Orthologs, paralogs, and evolutionary genomics.** *Annu Rev Genet* 2005, **39:**309-338.

57. Saeed AI, Sharov V, White J, Li J, Liang W, Bhagabati N, Braisted J, Klapa M, Currier T, Thiagarajan M, et al: **TM4: a free, open-source system for microarray data management and analysis.** *Biotechniques* 2003, **34:**374-378.

58. Tamura K, Peterson D, Peterson N, Stecher G, Nei M, Kumar S: **MEGA5: molecular evolutionary genetics analysis using maximum likelihood, evolutionary distance, and maximum parsimony methods.** *Mol Biol Evol* 2011, **28:**2731-2739.

59. Huang da W, Sherman BT, Lempicki RA: **Bioinformatics enrichment tools: paths toward the comprehensive functional analysis of large gene lists.** *Nucleic Acids Res* 2009, **37:**1-13.

60. Xu X, Liu X, Ge S, Jensen JD, Hu F, Li X, Dong Y, Gutenkunst RN, Fang L, Huang L, et al: **Resequencing 50 accessions of cultivated and wild rice yields markers for identifying agronomically important genes.** *Nat Biotechnol* 2012, **30:**105-111.

61. Guo S, Zhang J, Sun H, Salse J, Lucas WJ, Zhang H, Zheng Y, Mao L, Ren Y, Wang Z, et al: **The draft genome of watermelon (*Citrullus lanatus*) and resequencing of 20 diverse accessions.** *Nat Genet* 2013, **45:**51-58.

62. Zheng LY, Guo XS, He B, Sun LJ, Peng Y, Dong SS, Liu TF, Jiang S, Ramachandran S, Liu CM, Jing HC: **Genome-wide patterns of genetic variation in sweet and grain sorghum (*Sorghum bicolor*).** *Genome Biol* 2011, **12:**R114.

63. Jiao Y, Zhao H, Ren L, Song W, Zeng B, Guo J, Wang B, Liu Z, Chen J, Li W, et al: **Genome-wide genetic changes during modern breeding of maize.** *Nat Genet* 2012, **44:**812-815.

64. Kim HJ, Ono E, Morimoto K, Yamagaki T, Okazawa A, Kobayashi A, Satake H: **Metabolic engineering of lignan biosynthesis in Forsythia cell culture.** *Plant Cell Physiol* 2009, **50:**2200-2209.
